# Epigenetic control of PDX1 and NGN3 by a computationally designed PRC2 inhibitor enforces pancreatic endocrine differentiation from pluripotent stem cells

**DOI:** 10.21203/rs.3.rs-9115136/v1

**Published:** 2026-03-26

**Authors:** Laura Crisa, Shiri Levy, Dan Mar, Karol Bomsztyk, Stephanie Battle, Sina Gharib, Cecilia Lopez-Martinez, Patrick McGrath, Julie Mathieu, Marie Regier, Hannele Ruohola-Baker, Vincenzo Cirulli

**Affiliations:** 1 Department of Medicine, University of Washington, Seattle, WA, USA; 2 Department of Biochemistry, University of Washington, Seattle, WA, USA; 3 Department of Natural Sciences, Bowie State University, Bowie, MD, USA; 4 Department of Medicine and Computational Medicine Core, University of Washington, Seattle, WA, USA; 5 Charles C. Gates Center for Regenerative Medicine, University of Colorado School of Medicine, Anschutz Medical Campus, Aurora, CO, USA; 6 Department of Comparative Medicine, University of Washington, Seattle, WA, USA; 7 Department of Bioengineering, University of Washington, Seattle, WA, USA; 8 Institute of Stem Cells and Regenerative Medicine, University of Washington, Seattle, WA, USA.

## Abstract

Directed differentiation of pluripotent stem cells (PSCs) into pancreatic islets is a cornerstone strategy for diabetes cell therapy. This process relies on growth factor–driven activation of core transcriptional regulators, notably PDX1 and NGN3, to restrict the multi-lineage potential of definitive endoderm to pancreatic progenitors and endocrine cell types. Yet differentiation efficiency and lineage fidelity vary markedly across PSC lines. Here, we demonstrate that a dominant constraint is persistent Polycomb Repressive Complex 2 (PRC2)–mediated epigenetic repression at the *PDX1* and *NGN3* loci, limiting endocrine specification despite inductive signaling.

To directly test whether chromatin states at the *PDX1* and *NGN3* loci gate developmental competence, we deployed a computationally engineered epigenetic effector (EBdCas9) to transiently and sequentially remove H3K27me3 at those loci during defined developmental windows. Targeted epigenetic resolution robustly enhanced endocrine lineage commitment and accelerated β-cell differentiation across genetically diverse PSC lines. In contrast, direct transcriptional activation with VP64dCas9 increased PDX1 and NGN3 expression but did not improve differentiation outcomes. Integrated cell population studies and genome-wide chromatin and transcriptomic analyses reveal that PRC2-targeted remodeling preferentially activates endocrine gene networks while limiting progenitor expansion and lineage-inappropriate programs.

These findings establish that gene-targeted manipulation of PRC2-mediated repression at *PDX1* and *NGN3* can be used to control cell lineage competence. Collectively, our study reframes variability in PSC differentiation as a failure of epigenetic resolution rather than transcriptional insufficiency and introduces locus-specific chromatin remodeling as a generalizable strategy to enforce developmental fidelity.

## Introduction

The rapidly rising incidence and health burden of diabetes mellitus^1^ underscore the urgent need to address population-wide treatments that ideally overcome both organ-shortage and the unprecise metabolic control supported by conventional insulin therapy. In this regard, Stem Cell (SC)-based replacement of pancreatic islets has emerged as a promising therapeutic strategy^2–6^.

In SC cultures, production of islet cell lineages is directed by morphogens and inductive cues that recapitulate key stages of pancreatic organogenesis. This process proceeds through a sequential activation of transcriptional programs, first giving rise to definitive endoderm derivatives, then primitive gut tube and pancreatic progenitors, and ultimately endocrine progenitors that subsequently differentiate into hormone-producing cell types^5,6^. Along this developmental path, the transcription factors PDX1 and NGN3 govern two critical lineage decisions. Activation of the PDX1 locus is required for the specification of cells of the primitive gut tube into pancreatic cell lineages^7–9^. Downstream of PDX1, NGN3 expression directs pancreatic precursors with dual exocrine/endocrine potential to commit to the islets’ endocrine lineage^10–12^. Still, even under the most advanced protocol of directed differentiation, populations of pancreatic and endocrine progenitors generated from stem cell cultures remain highly heterogenous. Indeed, not all primitive gut tube cells become PDX1-positive during directed differentiation. In addition, although seemingly homogenous by PDX1 expression, only a fraction (10–20%) of these progenitors goes on to express NGN3 and NKX6.1 and engage into an endocrine differentiation pathway. This competence is highly variable across multiple stem cell lines^13–16^, frequently yielding heterogenous preparations containing inconsistent proportions of mature endocrine cells and immature or lineage-deviant cell types. Heterogeneity of SC-derived islet clusters at the endocrine progenitor stage was shown to condition the emergence of non-functional polyhormonal over mono-hormonal cell types, as well as the outgrowth of immature components into undesired cell types after transplantation, a noted safety risk of SC-based cell replacement therapies^16–21^.These observations highlight the inherent complexity of controlling the outcome of stem cell cultures relying solely on exogenous cues and inductive factors.

Epigenetic states of the chromatin, such as histone methylation and acetylation, are known determinants of SC fates, lineage specification and differentiation^22,23^. An epigenetic feature often found at the promoters of developmental regulators such as transcription factors is the co-existence of repressive H3K27me3 and activating H3K4me1 or H3K4me3 histone marks, creating “bivalent” chromatin states which poise developmental genes for activation once the repressive marks are removed^24,25^. By establishing and maintaining repressive H3K27me3 marks, endogenous methyltransferase subunits of Polycomb Repressive Complex 2 (PRC2) are key regulators of these bivalent chromatin states^26^. It was noted that while transition from endoderm to pancreatic progenitors *in vivo* is associated with progressive loss of repressive H3K27me3 marks at gene loci encoding for transcriptional regulators of endocrine fates^27,28^, this trend is only incompletely recapitulated in PSC’s directed differentiation, with many loci maintaining an inappropriately repressed state^29^. Increased signal and broader coverage of H3K27me3 marks at transcriptional start sites of “maturity genes” also characterizes juvenile vs adult beta cells^30^. These observations have pointed to a prominent role of PRC2 in restricting the activation of pancreatic lineage choices as well as regulating progression to maturity through the maintenance of locked chromatin states at key developmental and functional genes.

Structural studies have shown that PRC2 exists as a multi-protein complex where the Embryonic Ectoderm Development (EED) subunit acts as a stabilizer and enhancer of EZH1/2, the component of PRC2 endowed with histone methyltransferase activity^31^. Recent work has led to the development of a computationally designed mini-protein that binds to the EED interface of PRC2 with 300-fold greater affinity than its endogenous EZH2 binder^32^. By doing so, this EED binder, referred herein as EB, functions as a robust decoy that disrupts PRC2 complexes and their function. Importantly, when fused to catalytically dead Cas9 (dCas9) for gRNA-mediated gene targeting, EB is sufficient to activate the targeted gene by reducing the PRC2-dependent repressive histone modification H3K27me3^32,33^. Similar to other chromatin regulators^34–36^, gene-targeted EBdCas9 induces cell-autonomous spreading of chromatin remodeling beyond the targeted genes, leading to extensive deposition of activating H3K27Ac marks at neighboring loci and sustained epigenetic memory of gene activation even after transient delivery^33^.

Notwithstanding the dynamic changes of PRC2-dependent repressive marks noted during islet differentiation, direct probing of PRC2 functions at endocrine lineage-relevant loci in SC has not been attempted. Indeed, whether SC culture heterogeneity and ultimately yield of islet endocrine cells reflect insufficient remodeling of PRC2-dependent repressive chromatin states specifically at the *PDX1* and/or *NGN3* loci remains unexplored. Hence, in this study, we set out to investigate whether the deployment of EBdCas9 to *PDX1* and *NGN3* genes could be used to epigenetically modulate the transcriptional programs dependent on these transcription factors. We found that the transient, sequential targeting of selected promoter regions of *PDX1* and *NGN3* by EBdCas9 enforces pancreatic and endocrine lineages’ specification, ultimately accelerating the emergence of insulin-positive beta-like cells from stem cell lines of diverse genetic backgrounds and/or clonal origin, while significantly restraining development of lineage-inappropriate cell types. Genome-wide profiling of activated gene loci and transcriptomic signatures enriched under gene-guided EBdCas9 treatments further indicates a positive effect of the epigenetic intervention in activating gene networks critical to endocrine cell differentiation and secretory functions. These findings reveal the existence of PRC2-dependent epigenetic barriers to the activation of two master regulators of stem cells’ differentiation into pancreatic endocrine lineages, pointing to gene-targeted epigenetic modifiers as effective tools to accelerate differentiation, decrease the heterogeneity, and refine cell composition of islet tissue derived from directed differentiation of SC. More broadly, this programmable, locus-specific epigenetic remodeling strategy has the potential to be generalized to unlock lineage-defining regulatory networks in other developmental contexts, enabling the reliable generation of differentiated tissues from PSCs for regenerative medicine across a wide range of cell types.

## Results

### Derivation of hPSC lines allowing for conditional expression of the PRC2 inhibitor EBdCas9 during directed differentiation of pancreatic islet cells from definitive endoderm.

To investigate EBdCas9 epigenetic action in the context of islet cell differentiation, we engineered EBdCas9 into hPSC lines that would facilitate both the identification of Definitive Endoderm (DE), i.e. the earliest developmental bottleneck conditioning the efficiency of SC progression into pancreatic lineages, and the live monitoring of developing pancreatic β cells.

To generate PSC lines reporters of DE and β cell yields, we engineered the original hESC MEL1/Ins^GFP^ (Ref. 37) with a polycistronic construct FOXA2;T2A-nucBFP2 to target BFP at the endogenous FOXA2 locus (Fig. S1A). By this approach we generated double FOXA2^BFP^Insulin^GFP^ reporter lines, henceforth referred to as MEL1-Double^FOXA2/INS^ lines. Using a protocol of direct differentiation of PSC into pancreatic cell lineages^6^, we select clones of these lines generating up >95% FOXA2^+^ DE cells (Fig. S1B), which predictably yielded a sizable fraction of Insulin-producing GFP^+^ β cells (~30%) by day 35 of differentiation (Fig. S1C).

Next, one of these MEL1-Double^FOXA2/INS^ clones was engineered with a polycistronic Doxycycline (DOX)-inducible EBdCas9^mCherry^ construct (Fig. S1D) ^33^ into the safe-harbor AAVS1 site. Multiples clones of this new reporter line, termed MEL1-Double^FOXA2/INS^;EBdCas9^mCherry^, were then tested for their responsiveness to DOX. Figure S1E shows representative fluorescence microscopy images of the mCherry reporter effectively induced in most of the cells in two of such clones, after 24 hours of DOX treatment. Following the same approach, several DOX-inducible clones engineered with the EBdCas9 ^mCherry^ construct were generated from H1 hESC and CVI-A2 iPSC (Fig. S1F). qPCR analysis of the EB transcript expressed in these lines confirmed its transient expression upon DOX withdrawal (Fig. S1G). G-banding karyotyping and PCR-based genotyping demonstrated the lines harbored numerically and structurally normal chromosomes and carried the EBdCas9 ^mCherry^ construct correctly integrated in the AAVS1 site (Fig. S2A-C).

These newly generated stem cell lines with inducible EBdCas9 provide us with both tissue-specific and genetically diverse SC models for conditional induction and gene targeting of EBdCas9 during directed differentiation into pancreatic cell lineages.

### Targeting of EBdCas9 to *PDX1* and *NGN3* promoters induces gene transcription, along with loss of H3K27me3 repressive marks at the targeted genomic sites.

**Figure 1.**
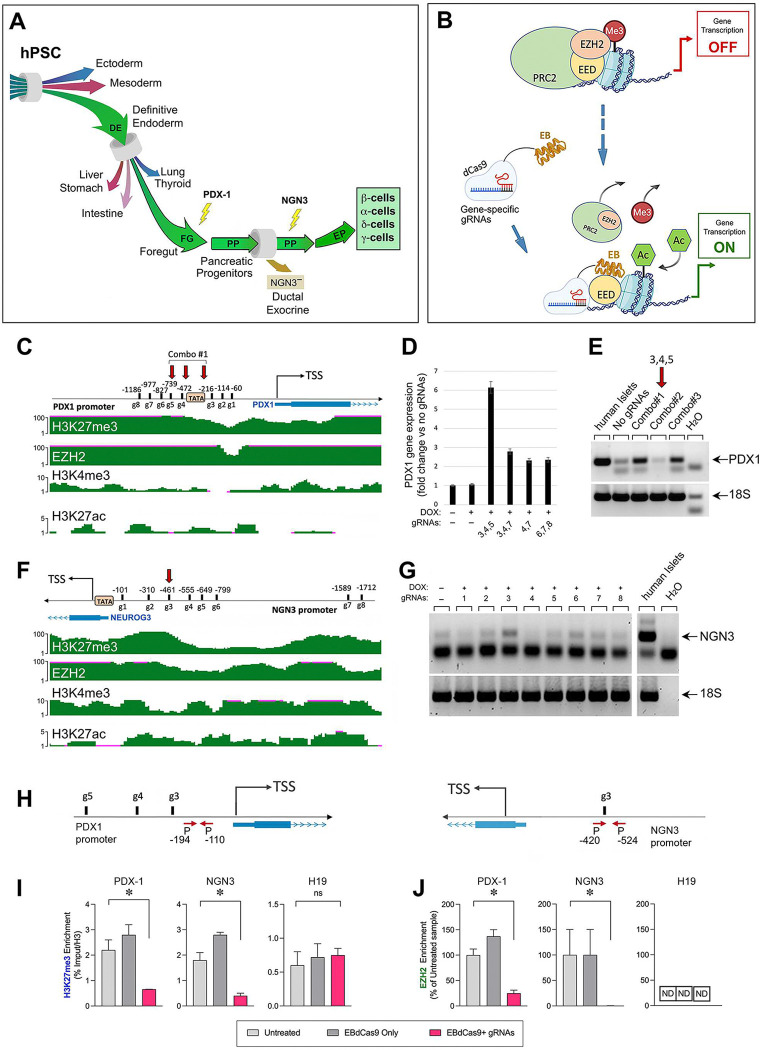
gRNA-mediated targeting of EBdCas9 to *PDX1* and *NGN3* promoters leads to gene activation and chromatin remodeling of the targeted genomic sites. (A) Bottlenecks in the differentiation of PSC toward pancreatic endocrine lineages. (B) Mode of action of EBdCas9. (C and F) Genomic coordinates of gRNAs designed for targeting the *PDX1* (C) and *NGN3* (F) promoters overlayed onto integrative genomic viewer of H3K27me3 marks and EZH2-binding sites of the corresponding genomic regions. (D, E, G) RT-qPCR analysis of PDX1 and NGN3 transcription detected 48 hours (PDX1) and 24 hours (NGN3) in MEL1-Double^FOXA2/INS^;EBdCas9^mCherry^ hESC after DOX induction of EBdCas9 alone or in combination with transfection of the indicated gRNAs. RT-qPCR of 18S of the same samples are shown as housekeeping controls. (H-I) Chip-qPCR analysis showing loss of H3K27me3 -marks and EZH2 occupancy in targeted *PDX1* and *NGN3* promoters. In contrast, non-targeted *H19* gene loci remain unaffected. Bars in I represent mean ± SD of H3K27me3 enrichment calculated as % of total input and normalized to total H3; Bars in J represent mean ± SD of EZH2 enrichment calculated as % of untreated samples. ND: not detected. *=P<0.05.

PDX1 and NGN3 transcription factors sequentially regulate pancreatic progenitor specification and endocrine lineage commitment (Fig. 1A). We hypothesized that competence to execute these two critical lineage choices could be facilitated by the de-repressing epigenetic action of EBdCas9 targeted to those gene loci (Fig. 1B). To investigate this possibility, we used ENCODE UCSC genome browser to inspect H3K27me3, H3K4me3 and EZH2 tracks of H1 hESC at promoter regions of *PDX1* and *NGN3*, ~ 1.5 kb upstream of the genes’ transcriptional start site (TSS). Within each promoter, we designed candidate targeting gRNAs proximal to predicted regulatory TATA boxes and/or specific to chromatin regions enriched for bivalent H3K27me3/H3K4me3 histone marks (Fig. 1C and 1F). For gRNA functional screening, undifferentiated EBdCas9 PSC were first incubated in serum-containing medium to allow the cells to exit pluripotency (day 1). On day 2 cells were then induced with DOX and on day 3 transfected with individual gRNAs, or combinations thereof, using lipofectamine. Controls (DOX only) were treated with lipofectamine only. RT-qPCR analysis of RNA extracted from these cultures 48 hours (PDX1) or 24 hours (NGN3) post-gRNA transfection, identified a cocktail of gRNAs (#3, 4, and 5, i.e., combo 1 (C1), Fig. 1D-E) and gRNA#3 (Fig. 1G), as most effective for the induction of *PDX1* and *NGN3* transcription, respectively.

Next, to analyze the epigenetic modifications induced by EBdCas9, samples of MEL1-Double^FOXA2INS^;EBdCas9 hESCs cultured and DOX-treated as described above, were harvested at 24, 48, and 72 hours post gRNA-transfection and processed for ChIP-qPCR. Chromatin immunoprecipitation using antibodies specific for EZH2 and H3K27me3 followed by qPCR of DNA regions flanking the genomic sites targeted by each gRNA, revealed significant reduction of EZH2 and depletion of H3K27me3 by day 3 post-transfection (Fig. 1H-J), indicating effective locus-specific displacement of EZH2 and progressive loss of histone repressive marks driven by EBdCas9. Importantly, untargeted loci such as the H19 Imprinting Control Region were not affected, supporting the specificity of the intervention (Fig. 1J). These results demonstrate that EBdCas9-mediated epigenetic regulation of islet-specific genes occurs at the intended loci and is efficient.

### EBdCas9/gRNA-mediated sequential activation of *PDX1* and *NGN3* promoters accelerates the development and increases the output of β-cells.

**Figure 2.**
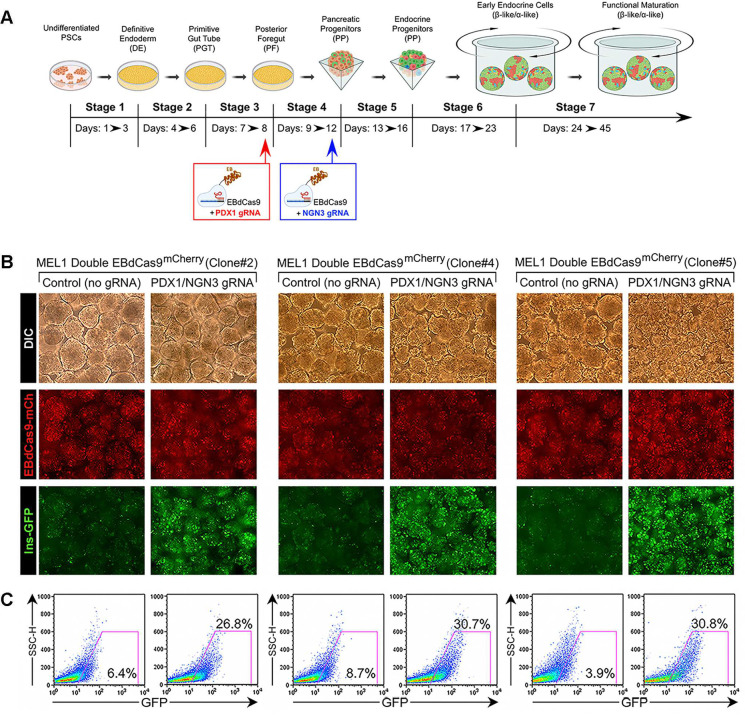
EBdCas9/gRNA-mediated sequential activation of *PDX1* and *NGN3* promoters accelerates the development and increases the output of β cells across multiple PSC clones. (A) Flow chart of EBdCas9 induction and gRNA delivery during directed differentiation of PSC toward pancreatic islet lineages. (B) Fluorescence microscopy of islet clusters from three MEL1-Double^FOXA2/INS^;EBdCas9^mCherry^ hESC clones at day 19 of differentiation showing cell expression of EBdCas9^mCherry^ and INS-GFP fluorescent reporters. (C) Representative flow cytometric analysis of GFP^+^Insulin^+^ cells from the samples shown in B.

To test the impact of EBdCas9-driven activation of *PDX1* in foregut cells, and *NGN3* in pancreatic progenitors on the generation of islet cells, we subjected three distinct clones of the MEL1-Double^FOXA2INS^;EBdCas9 cell line to directed differentiation toward pancreatic lineages. We used the protocol described by Balboa et al.^6^ modified to include induction of EBdCas9 at select time points (Fig. 2A). Briefly, at the end of “Stage 3” (Day 7 of differentiation), foregut cells were treated with DOX to induce EBdCas9 and next day (day 8) cells were transfected with a mix of the PDX-targeting gRNAs 3,4,5 (Fig. 2A). Subsequently, at the end of “Stage 4” (Day 11–12), the induction of EBdCas9 by DOX was repeated in pancreatic progenitor cells followed on the next day by transfection with NGN3-specific gRNA#3, identified as inducer of NGN3 transcription (Fig. 2A). Live monitoring of these SC cultures up to day 19 of differentiation revealed a striking higher number of Insulin/GFP^+^ cells in EBdCas9+PDX/NGN3 gRNA-treated cells as compared to control cultures treated with DOX alone without gRNAs (Fig. 2B). Validation of these results by quantitative flow cytometry revealed that all three PSC clones tested consistently exhibited a significant (~3 to 6-fold) increase in the fraction of β-cells, a cell yield normally observed much later at Stage 7 of differentiation (Fig. 2C).

To broaden the applicability of this epigenetic editing intervention to non-genetically manipulated PSC lines, we also tested the effects of delivering EBdCas9 via mRNA transfection together with gRNAs. Pilot experiments showed that transfection of Fluoro-labelled gRNAs resulted in successful gRNA incorporation in >85% of the cells (Fig. S3A). Delivery of mCherry-dCas9 mRNA via lipofectamine resulted in a significant shift in mean intensity of mCherry fluorescence (MIF) in most of the cells (MIF= 64±1.4 in mCherry-mRNA transfected cells vs 22 ±1.7 in lipofectamine only treated cells, mean ± SEM, n=4, P<0.0001; Fig. S3B), with a net ~40% mCherry^Hi^ cells calculated relatively to baseline autofluorescence of lipofectamine-only controls. Flow cytometric analysis of transfected cells at day 19 of differentiation showed significantly increased yields of insulin-positive cells in gRNA guided-EBdCas9 as compared to EBdCas9 only controls (i.e., mean ± SEM= 20.8% ± 2.8 in gRNA-guided EBdCas9 vs 5.3%±1.5 in EBdCas9 only, n=5 experiments, P<0.001) (Fig. S3C), recapitulating the effect observed in the engineered lines. Western blotting analysis of Cas9 protein expression in whole cell extracts of the transfected cells showed detectable levels of the guided-EBdCas9 up to 5 days post gRNA transfection (i.e., day 17 of differentiation), while expression of the non-guided construct was extinguished by day 3, and global levels of expression of EZH2 and H3K27me3 were maintained (Fig S3D-F). By this analysis, upregulation of PDX1 and NGN3 protein expression (~30–50% increase) was detected 4 days and 24 hours post gRNAs transfection, respectively (Fig S3G-H).

**Figure 3.**
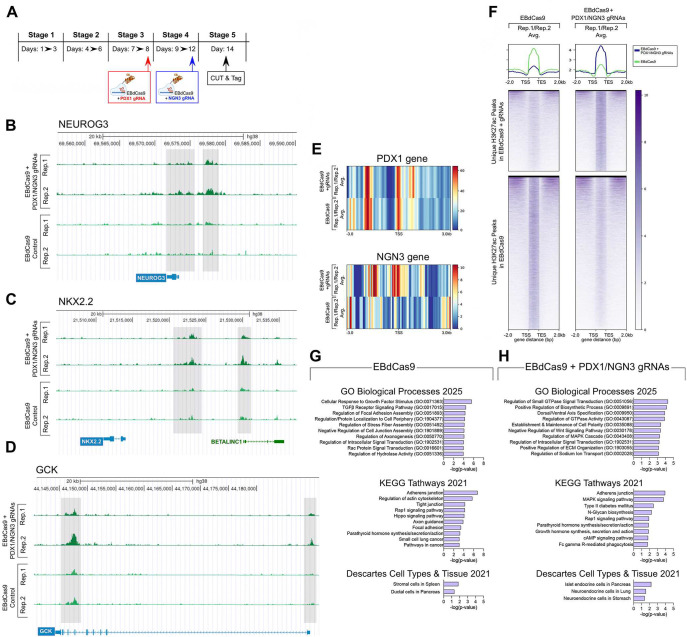
Sequential activation of *PDX1* and *NGN3* promoters by EBdCas9 leads to extensive remodeling of H3K27Ac chromatin modifications in differentiating PSC. (A) Timeline of EBdCas9 interventions during PSC differentiation and cell harvesting for CUT&Tag. (B-D) Genome browser view showing enhanced H3K27ac signal (shadowed) within the promoter, enhancer and/or intronic regions of NGN3, NKX2.2 and GCK, in PDX1/NGN3-guided EBdCas9-treated samples compared to control. Two biologic replicas (Rep) per condition are shown. (E) Heatmaps of H3K27Ac signal within the *PDX1* and *NGN3* gene loci. The averaged signal of the two biological replicas is shown. (F) Heatmaps of H3K27Ac signal showing unique peak calling of PDX1/NGN3-guided EBdCas9-treated samples (upper panels, 14695 peaks) and EBdCas9-treated controls (lower panels, 28033 peaks). For each condition, the top 5% significant peaks and averaged signal of two biological replicas are shown. Composite plots of normalized H3K27Ac signal are shown at the top of each heatmap. TSS: Transcriptional Start Site. TES: Transcriptional End Site. (G-H) Enrichment pathway and cell type analysis performed on the nearest neighboring genes to the top 1% unique H3K27Ac peaks, using the indicated gene data sets from Enrichr^41^. See also Tables S1-S6 for complete lists of annotated genes.

Previous studies combining ATACseq, ChiPseq and RNAseq data have shown that PSC’s competence to execute developmental transitions during directed differentiations toward islet lineages correlates with the early enrichment of H3K27ac histone modifications indicative of active chromatin states at select gene promoters and enhancers^38–40^. To investigate H3K27Ac signatures underlying the increased efficiency by which PSC cultures acquire endocrine phenotypes under gene-guided EBdCas9 interventions, duplicate chromatin samples from differentiating PSC induced to express EBdCas9 only or PDX/NGN3-gRNA guided EBdCas9 were processed for CUT&Tag and analyzed for genome-wide changes of H3K27Ac histone marks 48 hours after NGN3 gRNA transfection (day 14) (Fig. 3A). A total of 25.5 and 17.7 million sequencing reads were generated for H3K27Ac from control EBdCas9 only and PDX/NGN3-gRNA-guided EBdCas9 samples, respectively, which corresponded to 97% mapping of the human genome. Examples of H3K27Ac tracks detected in PDX/NGN3-gRNA-guided EBdCas9 samples showing enhanced H3K27Ac signals within the promoter regions of *NGN3*, as well as within promoter/enhancer regions of select genes target of *NGN3* (e.g., GCK and NKX2.2) and LncRNAs (e.g., Blinc1) are shown in Fig. 3B-D. Using a 5% cut-off threshold to identify significant peaks, 14695 and 28033 “unique” H3K27Ac peaks were found enriched in PDX/NGN3-gRNA-guided EBdCas9 and control EBdCas9 samples, respectively (Table S1-S2 and Fig. 3F). Heatmaps of these modifications spanning a 3Kb window centered on the TSS of the nearest neighboring gene to each peak, showed significant H3K27Ac enrichment at TSS-proximal regions and select distal intergenic regions (Fig. 3E-F). To explore enrichment of cellular pathways and phenotypes under each treatment, the nearest neighboring genes to the top 1% unique peaks (Tables S3-S4) were further analyzed using the Gene Ontology (GO) Biological process 2025, the KEGG pathways 2021, and Descartes cell types 2021 data sets from Enrichr^41,42^. Gene Ontology and KEGG pathway analysis indicated enrichment for genes involved in endocrine progenitors’ differentiation, including small GTPase-mediated signaling, Rap1 and cAMP signaling, establishment of apical/basal cell polarity, negative regulation of Wnt signaling and regulation of sodium Ion transport in PDX/NGN3-gRNA-guided EBdCas9 samples (Fig.3G, upper and middle panels). In addition, the topmost significant cell phenotype detected by the Descartes cell type data set aligned with pancreatic endocrine cells (Fig. 3G bottom panel). In contrast, H3K27ac histone modifications unique to EBdCas9 control samples were most significantly enriched for cell growth-related genes (e.g., cellular response to growth stimulus, TGFb-receptor signaling, Hippo signaling, and axogenesis) as well as pancreatic ductal and splenic cellular phenotypes (Fig. 3H, and Tables S5-S6).

Collectively, these results demonstrate that timed EBdCas9-mediated inhibition of the PRC2 complex at select *PDX1* and *NGN3* promoter regions accelerates progression of foregut cell types toward a robust specification of β-cell phenotypes. This outcome is triggered as effectively in transgenic and non-genetically manipulated SC lines through transient expression of EBdCas9, up-regulation of PDX1 and NGN3 transcription factors and activation of gene loci relevant to endocrine cell development and function at the expense of gene networks promoting cell growth and alternative non-endocrine fates.

### Distinct EBdCas9-mediated epigenetic interventions drive commitment of pancreatic progenitors to Endocrine Progenitor fates and endocrine lineage choices.

**Figure 4.**
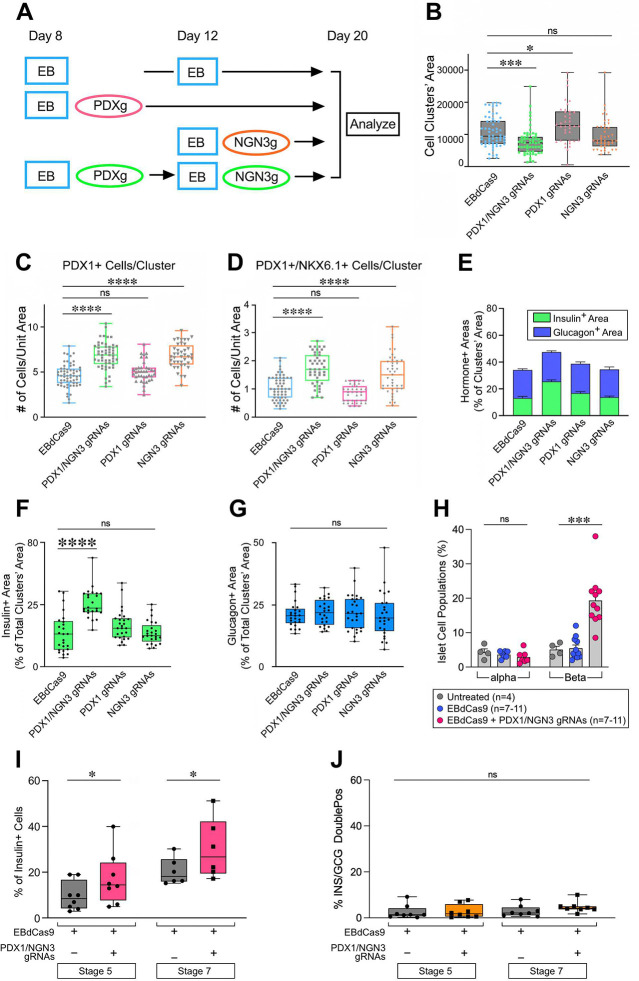
EBdCas9-mediated epigenetic interventions increase the efficiency of SC commitment to Pancreatic Endocrine Progenitors with developmental bias toward β cell types. (A) Schematic of EBdCas9 induction and gRNA transfection. (B-D) Morphometric analysis of SC clusters at day 20 of differentiation showing cell cluster areas (B), number of PDX1^+^ cells per clusters’ area (C), and number of PDX1^+^/NKX6.1^+^ Endocrine Progenitors per cluster’s area (D) under the indicated treatments. (E-G) Cumulative (E) and fractional representation of Insulin^+^ (F) and Glucagon^+^ (G) areas detected in the clusters at day 20 reveals increased yield of Insulin^+^ cells significantly induced by sequential targeting of EBdCas9 to *PDX1* and *NGN3* promoters. Box-and-whiskers-plots show the distribution of measurements in individual clusters. The thick line in each box represents the median. (H) Flow cytometric analysis of α and β cell subpopulations detected in multiple independent experiments using hESCs and iPSCs at day 20 of differentiation confirms the increased yield of β cell types under EBdCas9+PDX/NGN3 gRNA treatment. Bars are mean ± SEM (n=4–11). (I-J) Flow cytometric analysis of insulin single positive cells (I) and insulin/glucagon double positive cells (J) in follow-up experiments (n=6–8). Data reflects the analysis of 5 distinct PSC lines, including MEL1-Double^FOXA2/INS^ hESC (3 clones), H1 hESC and CVI-A2 iPSCs. Enrichment of insulin single positive β cells in EBdCas9+PDX/NGN3 gRNA-treated samples relative to EBdCas9 controls is detected at both stage 5 and stage 7 of differentiation (day 35). *=P<0.05; ****=P<0.001.ns= not significant.

To gain insights on the impact of *PDX1*- and *NGN3*-targeted EBdCas9 on the development of bi-potent pancreatic progenitors and endocrine cell populations, we performed immunofluorescence and morphometric analysis of pancreatic lineage markers and hormones (i.e. PDX1, NKX6.1, INS and GCG) on hPSC-derived clusters at day 20 and day 35 of differentiation. We examined samples induced to express EBdCas9 alone, EBdCas9 guided sequentially by PDX/NGN3 gRNAs combinations, as well samples treated with EBdCas9 guided with either PDX- or NGN3-gRNAs individually, as outlined in Figure 4A.

Assessment of cluster areas at day 20 of differentiation, revealed that samples treated with EBdCas9 guided sequentially by PDX1/NGN3 gRNAs were significantly smaller than those present in the other cultures (Fig. 4B). As compared to controls, these clusters comprised a significantly higher frequency of PDX1^+^ as well as a ~two-fold higher number of PDX1^+^/NKX6.1^+^ cell types (Fig 4C-D), marking foregut-derived cells successfully specified to the pancreatic lineage and committed to Endocrine Progenitors, respectively. Samples treated with EBdCas9 guided with NGN3 gRNA alone, but not those guided with PDX1 gRNAs only, had similar phenotypes, indicating a requirement for NGN3-guided EBdCas9 for most effective progression of hPSC toward Endocrine Progenitors’ fates. Interestingly, however, only samples treated with EBdCas9 guided sequentially by PDX1/NGN3 gRNAs were enriched for insulin-positive cells (Fig. 4E-F), whereas frequency of α cells identified by glucagon staining remained unchanged across all treatments (Fig. 4G). Flow cytometric analysis of differentiation experiments using multiple hESC and iPSC lines confirmed the enhanced efficiency and β cell developmental bias imparted by EBdCas9 guided sequentially by PDX1/NGN3 gRNAs starting at day 20 (Fig 4H) and continuing up to day 35 of differentiation (Fig. 4I). Frequency of insulin/glucagon double-positive cells remained low and were not significantly impacted by treatments (Fig. 4J).

To further investigate developmental programs activated downstream of EBdCas9’s action genome-wide, we performed bulk RNAseq of differentiated MEL and H1 PSC (day 20), induced to express EBdCas9, EBdCas9 guided sequentially by PDX1/NGN3 gRNAs or EBdCas9 guided by either PDX1 or NGN3 gRNAs as shown in Fig 4A. To identify downstream effects of the epigenetic interventions independent of line-specific genetic backgrounds, in these experiments we considered the two lines as experimental replicas, aiming at exploring transcriptomic changes concordantly regulated in both lines by gene-guided EBdCas9.

**Figure 5.**
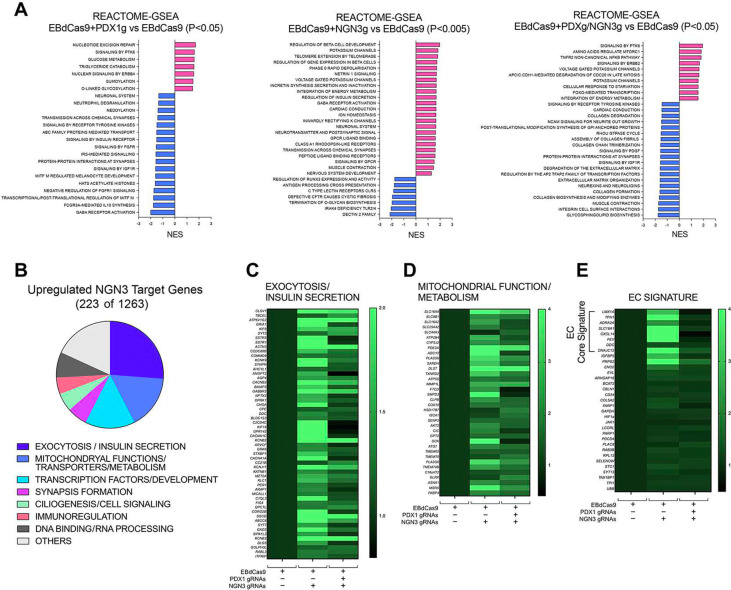
Sequential, EBdCas9-driven epigenetic remodeling of *PDX1* and *NGN3* promoters fosters the activation of NGN3-dependent transcriptional programs. (A) Gene Set Enrichment Analysis (GSEA) of transcripts, concordantly regulated in H1 and MEL1 hESCs differentiated to day 20, identifies differentially expressed Reactome Pathways in cultures of PDX1-, NGN3- and PDX1/NGN3-guided EBdCas9-treated samples as compared to EBdCas9 only control. (B) Fraction of predicted NGN3 target genes^43^ concordantly up-regulated in H1 and MEL1 hESCs epigenetically manipulated with EBdCas9+PDX/NGN3 gRNAs and differentiated to day 20, as detected by bulk RNAseq. Refer to Supplemental Table S10 for complete gene list and description. (C-D) Heatmaps of NGN3 target genes comprised within the Exocytosis/Insulin Secretion and Mitochondrial function/Metabolism categories shown in B. Heatmaps represent fold changes of normalized gene counts averaged in the indicated gRNA-treated vs EBdCas9 only H1 and MEL1 hESC samples. (E) Heatmap of Enteroendocrine (EC)-specific gene signatures. A gene signature strongly biased toward enteroendocrine lineages is detected in samples treated with EBdCas9-guided to *NGN3*-only promoter.

As compared to control EBdCas9 only, we identified 5105, 8047 and 6022 protein-encoding genes concordantly regulated across both cell lines in PDX1/NGN3-guided, NGN3-guided and PDX1-guided EBdCas9 samples, respectively. Gene Set Enrichment analysis (GSEA) of these concordant genes suggested that NGN3-guided EBdCas9 samples were positively enriched in molecular functions associated with early endocrine development and function (e.g., *Beta cell development, Voltage-gated Potassium channels*) and neuronal-related functions (e.g., *Neurotransmitter Receptors* and *Transmission across chemical synapsis*), whereas PDX1 and PDX1/NGN3-guided samples were positively enriched for metabolic functions (e.g., *Glucose metabolism, Triglyceride catabolism, FOXO-mediated transcription, Aminoacid regulation of mTORC1*), and negatively enriched for genes involved in neurotransmission and cell matrix interactions (e.g., *GABA receptors activation, Integrin signaling, Extracellular Matrix Organization* )(Fig. 5A and Supplementary Tables S7-S9).

We further compared the sets of concordantly up-regulated genes in NGN3- and PDX1/NGN3-guided samples to a published set of 1263 genes predicted and/or validated to be NGN3-bound in PSC-derived pancreatic progenitors^43^. From this analysis, we identified 223 NGN3 target genes concordantly up-regulated across NGN3-targeted EBdCas9 samples as compared to control (Fig. 5B and Table S10). The top three most represented classes of these genes were related to *Exocytosis/Insulin secretion*, *Mitochondrial function/Metabolism* and *Transcription Factors/Development*. Smaller gene sets were related to *Synapsis formation, Ciliogenesis, Immunoregulation, DNA binding and RNA processing* (Fig. 5B-D and Table S10). Intriguingly, among the gene sets related to development, we noticed transcription factors (e.g., LMX1A and PAX4) involved in the development of enteroendocrine-like cells^44^. Analysis of gene biomarkers previously linked to these endocrine populations^44,45^ revealed a core signature clearly up-regulated in the NGN3-targeted EBdCas9 but not in the PDX/NGN3-guided EBdCas9 samples (Fig. 5E), suggesting differential enrichment of this cell population in the two samples. Accordingly, immunostaining and morphometric quantification of Tyrosine Hydroxylase (TH)-positive cells within islet-like clusters at day 20 of differentiation revealed a significant increase in enteroendocrine-like cells in NGN3-guided EBdCas9 samples compared with both PDX1/NGN3-guided EBdCas9 samples and EBdCas9-only controls (Fig. S4A–C).

Taken together, these results provide evidence that targeting EBdCas9 solely to the *NGN3* promoter, or, sequentially, first to the *PDX1* and then to the *NGN3* gene, has distinct outcomes. In both interventions, EBdCas9’s action guided to the *NGN3* promoter is necessary and sufficient to enhance yields of Endocrine Progenitors and activate NGN3-driven transcriptional programs. However, downstream of Endocrine Progenitors, β over α and enteroendocrine lineage choices are dependent on EBdCas9-driven de-repression of the *PDX1* promoter prior to *NGN3* activation.

### EBdCas9- but not VP64-driven activation of *PDX1* and *NGN3* genes selects against uncommitted cells, enforcing pancreatic over gut developmental fates.

**Figure 6.**
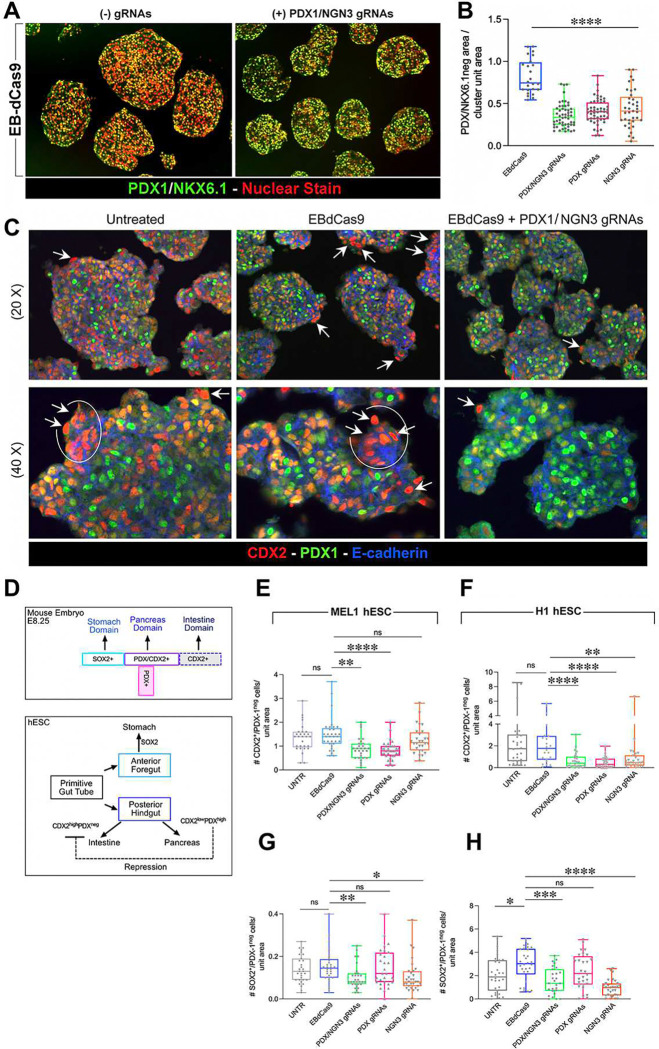
Gene-guided EBdCas9 select against uncommitted cells, enforcing pancreatic over gut developmental fates. (A-B) Representative fluorescence microscopy and (B) morphometric analysis of MEL1-Double^FOXA2/INS^ hESC clusters at day 20 of differentiation, showing the frequency of uncommitted PDX1/NKX6.1-negative cell types (red labelled nuclei) normalized to clusters’ areas under each treatment. (C) Uncommitted cells include PDX^neg^/CDX2^+^ gut epithelium (arrows and circles). (D) Schematic representation of gastric (SOX2^+^), pancreatic (PDX1^+^/CDX2^+^) and gut (PDX^neg^CDX2^+^) domains developing from primitive gut tube cells in mouse embryogenesis and during directed hESC differentiation toward pancreatic lineages. High levels of PDX1 are critical to repress CDX2 and intestinal fate choices at the Foregut/Hindgut boundary. (E-H) Morphometric analysis of PDX1^neg^/CDX2^+^ (E-F) and PDX1^neg^/SOX2^+^ cell types (G-H) counted per clusters’ areas under each at day 20 of directed differentiation of MEL1-Double^FOXA2/INS^ hESC (E,G) and H1 hESC (F,H). **P<0.01; ****P<0.001.

Mirroring the improved yields of pancreatic and endocrine progenitors described above, we found that all gene-guided EBdCas9 interventions resulted in significantly decreased numbers of uncommitted PDX1^neg^/NKX6.1^neg^ cells at day 20 of differentiation (Fig. 6A-B), indicating a positive effect of EBdCas9-driven gene activation in improving the recruiting of immature foregut progenitors into pancreatic developmental paths.

An alternative method to achieve CRISPR-mediated gene activation is through gRNA-guided dCas9 mediated delivery of synthetic trans-activators, such as VP64. Unlike EB, VP64 does not modulate the chromatin state, but rather functions as a scaffold for recruiting transcription factors of the preinitiation complex, thereby promoting gene expression^46^. To investigate whether this system would resolve the heterogeneity of islet clusters and improve β cells’ yield as we observed for EBdCas9, we engineered VP64 to replace EB fused to dCas9 in our mCherry reporter plasmid (Fig. S5A) and used this construct to synthesize VP64-dCas9-mCherry mRNA for cell delivery. Initially, to validate its function in inducing *PDX1* and *NGN3* transcription, this mRNA was used in transfections of undifferentiated iPSC to screen for gRNAs targeting PDX1 and NGN3 promoters’ regions proximal to the transcriptional start sites of each gene. The same PDX1-specific gRNA pool (i.e. gRNA 3,4,5) previously used for EBdCas9 and a new NGN3-specific gRNA were identified as effective at inducing VP64-mediated upregulation of PDX1 and NGN3 mRNA, respectively, as compared to VP64dCas9 mRNA treated controls (Fig S5B). Next, the VP64dCas9 mCherry mRNA alone or in combination with the selected PDX1- and NGN3-specific gRNAs were transfected into differentiating PSCs following the same timeline used for EBdCas9 (Fig. 4A). Western blotting analysis of cell extracts confirmed robust and sustained expression of dCas9 delivered by these transfections up to 7 days post NGN3 gRNA transfection (Fig. S5C-D). However, immunofluorescence and morphometric analysis of PDX1^+^ and NKX6.1^+^ cells at day 20 of differentiation showed that none of the gene-guided VP64dCas9 interventions increased the yield of PDX^+^NKX6.1^+^ Endocrine Progenitors nor significantly decreased the frequency of PDX1^neg^/NKX6.1^neg^ cells (Fig. S5E-G). In addition, morphometric analysis of insulin^+^ and glucagon^+^ cells at stage 7 in samples treated sequentially with PDX1 and NGN3-guided VP64dCas9 revealed no significantly enhanced yield of cells positive for either hormone (Fig. S5H-I). Hence, VP64-mediated activation and EB-driven epigenetic remodeling of *PDX1* and *NGN3* promoters are not functionally equivalent in driving downstream developmental decisions.

The positive effect of EBdCas9-driven interventions in reducing the number of uncommitted PDX1^neg^/NKX6.1^neg^ cells suggested the existence of PRC2-dependent epigenetic barriers to the transition from primitive gut tube to pancreatic lineages and raised the potential for cell drifting into alternative lineages. Alternative fates that PSC at the primitive gut stage could adopt are anterior foregut, marked by SOX2, and posterior mid/hindgut, marked by CDX2. CDX2 is a transcription factor transiently co-expressed with PDX1 in cells with dual intestinal/pancreatic developmental potential and thereafter remaining expressed at high levels in PDX1^neg^ cells committed to intestinal fates only^17,47–49^ (Fig. 6D). We therefore proceeded to investigate whether PDX1^neg^/NKX6.1^neg^ cells included populations expressing CDX2 and/or SOX2 at day 20 of differentiation. We found a significantly higher number of CDX2^+^/PDX1^neg^ cells in both untreated and unguided-EBdCAs9-treated clusters as compared to PDX/NGN3-guided EBdCas9 samples; CDX2^+^/PDX1^neg^ cells were Ecadherin^+^ and harbored larger nuclei than the rest of the cells in the clusters (Fig. 6C). Morphometric analysis of clusters differentiated from MEL1 and H1-hESC confirmed that PDX1-guided EBdCas9 as well as EBdCas9 guided sequentially to PDX and NGN3 promoters effectively selected against CDX2^+^/PDX1^neg^ cells in both lines (Fig. 6E-F). Untreated and EBdCas9-only treated clusters also contained rare SOX2^+^PDX^neg^ cells. These contaminants were significantly reduced in samples treated with EBdCas9 guided by NGN3 gRNA alone and were the lowest in samples that received sequential guidance with PDX1 followed by NGN3 gRNAs (Fig. 6G-H).

These findings demonstrate the ability of EBdCas9 interventions to enforce engagement of pancreatic over gut lineage choices during PSC directed differentiation toward islets cell lineages. Moreover, the data implicate PRC2-dependent repression of *PDX1* and *NGN3* promoters in differentially influencing posterior vs anterior gut developmental choices, respectively. Comparison with VP64-mediated gene activation systems further suggests that epigenetic modulation of *PDX1* and *NGN3* promoters directed by EBdCas9 is superior to forced transactivation of these transcription factors in improving differentiation efficiency and beta cell yields.

### Islet clusters epigenetically manipulated with PDX/NGN3-guided EBdCas9 are glucose responsive *in vivo*.

**Figure 7.**
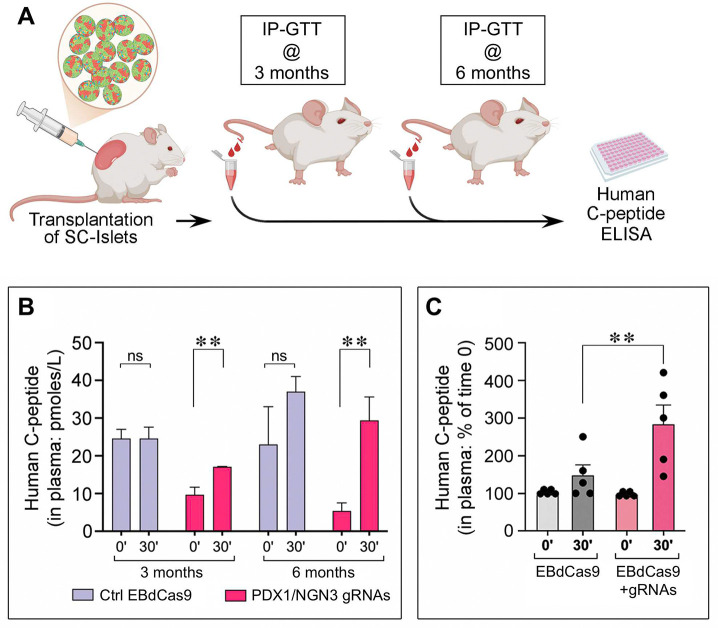
Stage 7 islet clusters epigenetically modified by PDX/NGN3-guided EBdCas9 are glucose responsive *in vivo*. (A) Time course of *in vivo* experiments. (B) Levels of human C peptide measured in IL2Rγ-−/− SCID mice in response to intraperitoneal glucose tolerance tests (IPGTT, 2mg/kg after 5 hours fasting) after transplantation of stage 7 islet clusters. Bars are mean ± SD of n=2 experimental mice per group, tested sequentially at 3- and 6-months post-transplantation. * P<0.05. (C) Human C peptide detected in 5 independent experiments during IPGTT performed 3–6 months post-transplantation, showing significant increased insulin secretory responses over basal secretion in mice transplanted with islet clusters resulting from treatments with PDX/NGN3-guided EBdCas9. Transplanted clusters were generated from differentiations of 3 distinct MEL1-Double^FOXA2/INS^ hESC clones. **P<0.01, ns= not significant.

To compare the ability of PDX/NGN3-guided EBdCas9 and EBdCas9 only control samples to respond to glucose *in vivo*, about 500 cell clusters at Stage 7 of differentiation were transplanted under the kidney capsule of immunodeficient NSG mice (Fig. 7A). Sequential measurements of circulating human-C peptide in response to intraperitoneal GTTs (IP-GTT) at 3 and 6 months after transplantation revealed a consistently lower basal insulin secretion at fasting and increased insulin secretion in response to glucose already at 3 months post-transplantation in mice carrying the epigenetically edited cells (Fig. 7B). In contrast, mice carrying EBdCas9-only treated grafts exhibited higher basal insulin secretion at fasting at all time points and variable responses to glucose loads at 6 months post-transplantation (Fig. 7B). In experiments using 4 distinct PSC lines, stimulation indexes (i.e., ratios of C-peptide secreted after glucose load over that measured at fasting) measured at 3–6 months post-transplantation, were significantly higher in mice carrying epigenetically edited SC-islets than in controls (Fig. 7C). Hence, the reportedly immature endocrine features of PSC transplants^6,16^—namely high basal insulin secretion and a blunted response to glucose—are resolved more rapidly and across genetically diverse backgrounds in epigenetically manipulated PSC samples.

## Discussion

The use of human pluripotent stem cells (PSC) is rapidly emerging as renewable source of patient-specific cell types for replacement therapies, disease modeling and drugs screen. All these applications, however, require the ability of PSC to differentiate into disease-relevant cell types, a competency that is highly variable among lines of different genetic backgrounds, origin or methods of derivation (e.g., hESC or iPSC)^50,51^.

In directed differentiation of PSC toward pancreatic islet lineages, protocols are frequently limited by heterogeneous and inefficient specification of pancreatic and endocrine progenitors. Variability in the formation of these intermediate populations propagates through downstream developmental stages, constraining both the yield and functional maturation of endocrine cell types—most notably insulin-producing β-cells. To overcome this bottleneck, early studies have attempted forward programming by overexpression of transcription factors such as *PDX1* and *NGN3*, known master regulators of those developmental stages. Results from these approaches have highlighted several challenges. For example, constitutive overexpression of PDX1 in mouse ES cells was shown to enhance the number of exocrine and endocrine progenitors indiscriminately, ultimately with no significant improvement to the number of insulin-positive cells^52^. In another study, overexpression of PDX1 in differentiating PSC via mRNA delivery resulted in a detectable increase of insulin transcription only after multiple transfections^53^. These results underscore the challenges associated with transcription factors’ overexpression as these approaches likely fail to achieve the precise gene dosage, temporal and/or cell-specific regulation required for the intended developmental effects.

In the present study, we demonstrate that epigenetic control of *PDX1* and *NGN3* transcriptional programs in PSC can be achieved by gene-targeting of the PRC2 inhibitor EBdCas9. We show that select chromatin regions of *PDX1* and *NGN3* promoters are sensitive to this synthetic epigenetic modifier, resulting in clearance of repressive H3K27me3 histone marks at the targeted sites and effective priming of those genes for activation. During directed islet cell differentiation, deployment of EBdCas9 to *PDX1* or to *NGN3* promoters, alone or in sequence, has distinct non-overlapping outcomes. In line with the known developmental roles of PDX1 and NGN3 reported by gene knock-out studies^9–11,54^, EBdCas9-driven activation of *PDX1* and *NGN3* significantly enhances yields of pancreatic PDX1^+^ progenitors and PDX1^+^NKX6.1+ cells endocrine progenitors, respectively. However, neither intervention alone impacts the α/β endocrine fates of those precursors. Instead, accelerated production and improved yields of β, but not α, cells are observed only if the two promoters are both targeted sequentially. These results are consistent with reported reciprocal and autoregulatory loops described for PDX1 and NGN3^55,56^, and more recent evidence for pioneering functions of NGN3^57^. Accordingly, mapping of active chromatin states reveals enhanced H3K27ac signal at the promoter and enhancer regions of *PDX1*, *NGN3* as well as *NGN3* target genes (Fig.3B-E). The β cell developmental bias induced by PDX/NGN3-guided EBdCas9 interventions may reflect a direct role of active *PDX1* on sustaining β cell identity^60^ or downstream activation of certain *PDX1* and *NGN3* target genes (e.g., *PAX4*, a known inducer of β cell phenotypes) ^58,59^. In the mouse, it was also shown that timing of *NGN3* activation and related patterned chromatin states may play a role in preferentially stirring endocrine progenitors toward β cell fates^61,62^.

Genome-wide profiling of H3K27ac histone modifications unique to the epigenetic intervention further suggests early activation of gene networks critical to endocrine differentiation, such as genes involved in the regulation of oxidative metabolism, establishment of apical/basal cell polarity and inhibition of WNT signaling. The latter is particularly noteworthy, as WNT inhibition has been previously shown to modulate the ratio between progenitors and endocrine cells in developing SC-clusters^63,64^. In view of the reportedly variable, cell line-specific, responsiveness to pharmacologic WNT inhibitors in SC differentiation protocols, and their potential to indiscriminately target Wnt4 and 5 signaling required for endocrine secretory functions^63–67^, our finding suggests that EBdCas9’s epigenetic action could be used to foster the engagement of WNT inhibitory pathways in a cell-autonomous, self-regulated, manner.

Overall, these findings reveal the existence of multiple early epigenetic barriers to the progression from pancreatic progenitors to hormone-positive β cells, highlighting the benefits of chromatin priming in directing cell fates dependent on *PDX1* and *NGN3* activities and the limitations of exogenous cues provided in culture to sustain those activities.

Beyond improved yields of PDX^+^/NKX6.1^+^ endocrine progenitors and accelerated generation of β cells, we found that PDX/NGN3-guided EBdCas9 interventions select out alternative CDX2/SOX2 gut derivatives as well as enteroendocrine cell types in stem cell cultures. Lingering of CDX2^+^ and SOX2^+^ transcriptional signatures in SC culture past pancreatic progenitor stages has been noted in previous transcriptomic studies^16,17,68^, and outgrowth of gut-related teratomatous tissue has been reported in transplants^19–21^. Interestingly, we found that the activation of either PDX1 or NGN3 transcriptional programs had different effects on limiting gut-related alternative fates. Specifically, we observed that samples treated with PDX1-guided EBdCas9 were significantly depleted of PDX^neg^/CDX2^+^ cell types, marking posterior foregut cells failing to engage into pancreatic developmental fates. Previous studies have reported on the requirement for PDX1 to rise to a critical threshold in order to suppress CDX2 in foregut cells and promote pancreatic organogenesis^49^. In PSC cultures, the outgrowth of PDX^neg^/CDX2^+^/NKX6.1^neg^ cells can be controlled by protocol-tailored BMP inhibitors but only with variable efficiency^69^. Based on these observations, our finding supports the idea that, under growth-factor directed differentiation, PDX1 insufficiency imparted by the epigenetic state of the gene is a critical variable contributing to progenitors’ drifting into gut derivatives. Conversely, we found that NGN3-guided EBdCas9 interventions, alone or in combination with PDX1-guided EBdCas9 treatment, were associated with depletion of PDX^neg^ progenitors expressing SOX2, a pluripotency gene reportedly expressed at stage 5 of SC differentiation^17^ and remaining highly expressed in the anterior foregut. In neuronal lineages, there is evidence that effective neurogenesis requires SOX2 repression by a NeuroD1-dependent negative feedback loop activated by NGN1/2^70^. As NeuroD1 is a direct target of NGN3 as well^43^, it is possible that a similar feedback loop governs endocrine specification in islet lineages.

Our transcriptomic analysis of NGN3-guided EBdCas9 treatments further reveals enrichment for gene sets involved in endocrine secretory, mitochondrial and synaptic/neuronal functions, many of which are reported targets of *NGN3*^43^. Curiously, PDX1-guided EBdCas9 intervention prior to NGN3 targeting was associated with attenuation of some of these traits, particularly of neuronal-like phenotypes. Several independent studies have reported on enhanced neuronal traits of SC-derived β cells and fetal islets relatively to adult β cells^71–73^, thereby linking those traits to cell immaturity. Gene sets encoding for such neuronal traits are uniquely devoid of H3K27me3 repressive marks in developing β-cells^28^. Our findings show that selective remodeling of PRC2-dependent repressed states of the NGN3 promoter is a strong driver of neuronal β cell phenotypes, whereas the resolution of these traits and progression to maturity may depend on sustained active chromatin states of *PDX1*.

Another striking effect of PDX1-guided EBdCas9 intervention prior to *NGN3* targeting is the downregulation of enterochromaffin-like transcriptional signatures, which we show results from an actual depletion of enterochromaffin-like cells from the clusters. Notably, similar enterochromaffin (EC) like-cell populations have been reported in the human fetal pancreas^71^. These cells appear to arise from PDX1^+^/CDX2^+^ progenitors capable of branching-out to either β-like or EC cell types depending on CDX2 expression^71^. Considering these observations, our findings point to PRC2-regulated PDX1 activation as a key checkpoint influencing CDX2-driven lineage decisions at both the gut-to-pancreatic and EC-to-β cells branch points. While TH^+^ enterochromaffin-like cells appear to be a common byproduct of differentiating foregut derivatives^17,45,73,74^, there is controversy as to whether the presence of these cell types in transplant settings contributes to the delay in glucose responsiveness of PSC-derived islet grafts observed *in vivo*, or even a safety risk due to their plasticity and proliferative potential^17^. To address these concerns, cell sorting approaches have been tested^17,75,76^ but the scalability of these strategies remains uncertain. Our findings indicate that epigenetic regulation of *NGN3* and *PDX1* gene activities may be an alternative approach to control the development of enteroendocrine cells during directed SC differentiation.

One last notable effect of PDX1/NGN3-guided EBdCas9 interventions is the tighter control of basal insulin secretion shown by cell grafts both at 3- and 6-months post-transplantation. This contrasted with heightened insulin secretion observed at fasting in control grafts, an issue reported by others at early time points post-transplantation and linked to decreased glucose thresholds in immature β cells^77^. Although a similar number of clusters were transplanted, the smaller size and different cell composition of epigenetically manipulated clusters vs controls, made it difficult to compare absolute levels of insulin secretion measured in response to glucose stimulation. A common approach to normalize for variation in cell composition, is to compare the “stimulation index” among experimental groups, i.e. the ratio of C-peptide measured in response to high glucose loads over C-peptide measured at fasting. We found that stimulation indexes of epigenetically manipulated clusters was significantly higher than that of controls across multiple experiments using different PSC lines. Hence, the epigenetic manipulation tested here is compatible with glucose responsive islet secretory functions across genetically diverse backgrounds.

Some limitations to these studies remain. Different global levels of H3K27me3 were shown to define specific β cell subtypes^78,79^. Our analysis does not address whether PDX/NGN3 gene-targeted EBdCas9 changes endocrine progenitor pools leading to the emergence of select α or β cell subtypes. Timed scCUT&RUN assays combined to scRNAseq may be needed to resolve epigenetic changes induced by EBdCas9 at the level of select progenitor populations and trace developmental trajectories associated with those changes to distinct endocrine cell types and subtypes.

Unlike gene-guided EBdCas9, VP64dCas9 targeted to the same chromatin regions failed to enhance β-cell yield, despite comparable induction of *PDX1* and *NGN3* transcription. These results indicate that, in the context of those two gene loci, the PRC2 remodeling action of EBdCas9 rather than merely “gene activation”, is most critical to drive pancreatic lineage commitment and enhance β-cell output. Differences in the stability of EB- and VP64-dCas9 (Fig S3E and S5C) may play a role in these differential effects. Alternatively, the two interventions may be differentially permissive to the maintenance of autoregulatory loops, oscillatory dynamics and/or endogenous transcriptional regulators inherent to *PDX1* and *NGN3* promoters^40,80,81^, which may be critical to sustain downstream developmental effects. Evidence that synthetic trans activators may sterically hinder the binding of endogenous transcriptional regulators at gene regulatory regions has been reported^82^. Further studies are warranted to discern these possibilities.

In conclusion, our findings provide novel evidence that, beyond line-specific optimization of growth factor-directed protocols, the efficiency and specificity of islet-lineage differentiation from diverse PSCs depend on controlled release of PRC2-dependent repressive mechanisms of the *PDX1* and *NGN3* genes. We show that EBdCas9 is an effective tool to manipulate PRC2 repression at select loci, offering a precise gene-targetable approach to accelerate differentiation and decrease the heterogeneity of PSC-derived islet cells for cell therapies, and, possibly, regenerative interventions^83^. For disease modeling applications, the use of epigenetic modifiers such as EBdCas9 could be further exploited to define the pathogenic role of altered epigenetic states of select loci in diabetes, as guided by ongoing genome-wide chromatin studies^84,85^.

## Materials and Methods

### Derivation of human PSC reporter lines of pancreatic islet differentiation.

We used CRISPR/Cas9 technology and homology-directed recombination (HDR) to engineer a polycistronic construct FOXA2;T2A-nucBFP2 targeting BFP at the endogenous *FOXA2* locus of the original hESC MEL1/Ins^GFP^ (kindly provided by Dr. Stanley)^86^. The targeting construct consisted of a bicistronically (T2A) expressed mTagBFP2 (FOXA2), immediately followed by a loxP-flanked Puromycin resistance cassette. It was synthesized with 1kb homology arms from the target locus, such that Cas9-mediated integration occurs in-frame with the target gene downstream of the full open reading frame and immediately 5’ of the endogenous stop codon. Guide RNAs were designed using the CRISPOR design tool. A guide RNA targeting the FOXA2 locus was synthesized by Synthego as single guide RNAs (sgRNAs) with the following sequence: GAAGCCGTCGTCTTCTTAAG.

PSCs were transfected using the P3 Primary Cell kit on a Nucleofector 4D unit (Lonza V4XP-3012), 1ug of Cas9 mRNA (Tri-Link), 2ug of synthesized sgRNA, and 1ug of plasmid. Cells were cultured under Puromycin selection for 14 days. The Puromycin cassette was then excised using Cre mRNA (Tri-Link) transfected into the hPSCs using RNAiMax (Thermofisher) following the manufacturer instructions. PCR of the insertion site, followed by Sanger sequencing, was used to confirm the fluorescent reporter was integrated in-frame. For sequencing we used a FOXA2-specific forward primer (5’- GAGCTGAAGGGGACGCCG-3’) and a mTagBFP2 - specific reverse primer (5’ ACTCCGCCATCTTCATACGT). The transfected hPSC pool was clonally expanded following single-cell deposition into 96-well plates using a Sony MA900 cell sorter. Clonally expanded lines were screened for puromycin sensitivity to confirm excision of the selection cassette. Cell clones were further quality controlled for appropriate up-regulated expression of BFP and GFP expression at the DE and EP stages of PSC differentiation, respectively, as measured by fluorescence microscopy and flow cytometry.

### Engineering of EBdCas9 in the AAVS1 site of hPSC lines.

MEL1-Double^FOXA2/INS^ hESC as well as H1 hESC and CVI-A2 iPSC were engineered with a polycistronic Doxycycline (DOX)-inducible EBdCas9^mCherry^ construct^33^ targeted to the AAVS1 safe-harbor site using the AAVS1 site-specific gRNA GGGGCCACTAGGGACAGGAT. In the targeting construct, EB is fused to dCas9-NLS-2A-mCherry plasmid^33^ with a 30 aa residue 6×5 (SGGGG) linker under control of the AAVS1-TREG inducible promoter. Nucleofection of MEL1-Double^FOXA2/INS^ hESC with targeting plasmid, gRNA and CAS9 mRNA followed the same strategy outlined above. For EBdCas9 targeting into H1 and CVI-A2 PSC, we used TALENS-targeting plasmids (Addgene #59025 and # 59026) to enforce recombinant homology at the AAVS1 site on chromosome 19. Clones of H1;EBdCas9 and CVI-A2;EBdCas9 PSCs were hand-picked under sterile conditions, expanded and banked. Validation of the correct insertion of the transgene included verification of mCherry expression following DOX treatment by flow cytometry, and genotyping by PCR using forward and reverse primers binding upstream and downstream of the 5’ and 3’ homology arms of the transgene^87^. The nucleotide sequence of the primers was: F1: TCGACTTCCCCTCTTCCGATG; R1: CTCAGGTTCTGGGAGAGGGTAG; R2: GAGCCTAGGGCCGGGATTCTC. The F1/R2 primer set was used to detect the insertion of the construct into AAVS1 locus (PCR amplicon: 1.2kb). The F1/R1 primer set was used to detect unmodified AAVS1 (PCR amplicon: 1.4kb). The co-amplification of the 1.2-and 1.4-kb fragments was used to identify mono-and biallelic insertion at the AAVS1 locus. Chromosome banding and karyotyping was performed by Diagnostic Cytogenetics, Inc. (Seattle, WA, USA).

### Guide RNA design, EBdCas9- and VP64dCas9-mCherry mRNA synthesis and transfection.

To identify sequences suitable for targeting of EB- and VP64-dCas9 to the promoter regions of PDX-1 and NGN3, we used ENCODE UCSC genome browser to inspect H3K27me3 and EZH2 tracks of H1 hESC within the ~1.7 Kb promoter region proximal to the transcriptional start site of each transcription factors’ gene. For each gene, sets of 8–9 guides were synthesized (Synthego). The nucleotide sequence and genomic distance from the TSS of the screened gRNAs is shown in Supplemental Tables S11A and 11B.

Full length EBdCas9-mCherry mRNA and VP64dCas9 mCherry was synthesized by Trilink Biotech using the sequence shown in Tables S11C and S11D. mRNAs were modified with full substitution of N1-Methyl-Pseudo-U Capped (Cap 1) and purified using CleanCap^®^ M6 Polyadenylated (120A) DNase Treatment, Oligo dT Purification, and elution in 1 mM Sodium Citrate, pH 6.4 solution. mRNA sequences are provided in Supplemental Table S11C.

For screening of gRNAs, iPSC;EBdCas9-mCherry were cultured to ~50–60% confluency in either DMEM-10% FCS (PDX1 promoter screening) or TeSR (NGN3 promoter screening) in 6 wells plates, treated with DOX (2 ug/ml) for 24 hours and then transfected using 2 ug of each gRNA and 10ul of lipofectamine RNAiMax transfection reagent in 250 ul of Opti-MEM. For cell transfection with both gRNAs and EBdCas9 or VP64dCas9-mCherry mRNA, 3ug of the purified dCas9-mCherry mRNA and 1ug of gRNAs were incubated in 125ul Opti-MEM together with 5ul of Lipofectamine MessengerMax transfection reagent (Invitrogen, LMRNA003) for 7 minutes and transfected onto 2 ml of cell suspension per well of a 6 well plate. Forty-eight hours after transfection, cells were harvested and processed for RNA extraction and RT-qPCR analysis.

### Chromatin immunoprecipitation and ChIP-qPCR.

MEL1-Double^FOXA2/INS^ hESC (300 X10^6^/well of a 6 well plate) were plated in TeSR in the presence of DOX (2ug/ml). The next day, the medium was replaced with DMEM-10% FCS plus DOX (2 ml/well) and cells transfected with PDX-1- or NGN3-specific gRNAs using 1 ug of each gRNA and 10ul of lipofectamine RNAiMax transfection reagent in 250 ul of Opti-MEM. 24–72 hours after transfection, cells were harvested using TrypLE, blocked in medium and pelleted by centrifugation at 1400 rpm. Cell pellets were washed with PBS and cross-linked by adding 500ul 1% formaldehyde in PBS for 20min at RT. After centrifugation, cell pellets were resuspended in 500ul of PBS/glycine (125mM) and incubated for 5 min at RT for quenching. Supernatant was removed and the cells were washed with 500ul of PBS. PBS was removed and samples were stored at −80°C. For shearing, cells were resuspended in 100ul chromatin sheering buffer (Active Motif), transferred into wells of a 96 well plates and placed in PIXUL for shearing. PIXUL parameters were as follows: Cycles= 50; PRF= 1kHz; Burst= 20 for 6min × 4. Sheered chromatin samples were added to a UV-treated polypropylene 96-well microplate in blocking buffer ( 150 mM NaCl, 50 mM Tris-HCl (pH 7.5), 5 mM EDTA, NP-40 (0.5% vol/vol), Triton X-100 (1.0% vol/vol), 5% BSA, sheared salmon sperm DNA (10μg/μL final)) and incubated in ultrasonic bath for 60 min at 4°C in the presence of Matrix-ChIP antibodies. A duplicate set of wells were prepared by coating the plastic with protein A and blocked in the same blocking buffer. The blocking buffer was aspirated from the protein A-coated plate, and the chromatin + antibody mix from the first set of wells was transferred to the protein A-coated wells and incubated in the ultrasonic bath for 60 min at 4°C. The wells were then washed 3 times with immunoprecipitation buffer followed by 3 washes with TE buffer. Finally, elution buffer containing 25 mM Tris base, 1 mM EDTA (pH10) with proteinase K 200 μg/mL was added to the wells, centrifugated for 30 s at 1400 rpms and incubated for 45 min at 55°C and then 10 min at 95°C. After mixing, the 96-well plates were centrifuged for 3 min at ~  500g at 4°C and used for PCR. The antibodies used for Matrix ChIP were: H3K27me3 (Active Motif 39155), EZH2 (Cell Signaling D2C9), H3K27ac (Active motif 39133) control Mouse or Rabbit IgGs (Vector Lab). Matrix ChIP experiments were performed in triplicate followed by qPCR in 4–8 replicates. Primers used for ChIP-qPCR were: PDX-1 Fw: CGTTCAGGAGTGTGCAGCAA; PDX1-Rev: CTAAGAGGCTAGGCCCAGGT; NGN3 Fw: CGCACAGGAAGATAGTGGCA; NGN3 Rev: GAGCAGGGCGTCCTTTAGAA; H19-ICR Fw: GAGCCGCACCAGATCTTCAG; H19 ICR Rev: TTGGTGGAACACACTGTGATC.

### PSC lines maintenance and differentiation into islet cell types.

PSCs were routinely maintained by culturing on Cultrex-coated dishes (diluted 1:30 in medium) in TeSR^Plus^ (Clonetics) in a 5% CO2, 20% oxygen incubator. Medium was changed every 24 hours. When cells reached ~80% confluency, cells were passed with TrypLE and replated onto fresh Cultrex-coated plates in the presence of 10uM ROCKi for the first 8–16 hours and used in differentiation experiments up to 15–20 passages.

To differentiate these lines into islet cell types, we followed a seven-stage differentiation protocol modified from our previous studies^5^ and adapted from Balboa^6^ and Rezania^3^. Stage 1 through stage 7 culture media and additives were exactly as described by Balboa et al. Cultures were started by lifting PSC with TryplE, blocking in culture medium and replating 1.5–2×10^6^ PSC/well on Cultrex-coated 6 well plates in 3 ml of TeSR^Plus^ medium in the presence of 10uM ROCKi (day 0). The next day, stage 1 culture medium was applied. At day 11, cell monolayers were lifted by TrypLE treatment, blocked, counted and replated at a cell density of 1.5–2×10^6^ in 6-wells Aggrewells (Stem Cells Technology), in 4 ml of the appropriate stage medium. Prior to cell replating, Aggrewells were treated with anti-adherence cell rinsing solution (Stem Cell Technology cat#07010), centrifuged at 2,400 RPMI to eliminate air bubble, and washed x 3 with PBS. After cell seeding, plates were centrifuged at 1,400 RPMI for 7 minutes, and then carefully moved to a 5% CO2, 20% oxygen incubator. At day 15 of differentiation, the cell aggregates were transferred to cell suspension culture plates in 5 ml of the appropriate stage medium and placed on a rotating platform at 95 RPMI until the end of differentiation (Stage 7). From Stage 1 to 6, all media and supplements were replaced every 24 hours. At stage 7, culture medium was replaced every 48 hours.

To enforce PDX1 and NGN3 transcriptional programs using EBdCas9-engineered lines, the differentiation protocol was modified at select stages. Specifically, for induction of *PDX1*, cells at day 8 of differentiation were treated with DOX (2μg/ml) to induce EBdCas9. The next day, cells from each 6 well were lifted with TrypLE and replated in the appropriate culture medium in the presence of 10uM ROCKi and DOX (2μg/ml) on a fresh Cultrex-coated 6 well in 2.5 ml of medium; 125 microliters of Opti-MEM medium containing a combination of PDX-1-specific gRNAs (gRNA# 3,4,5, 1μg/each) and 5ul of lipofectamine RNAiMax transfection reagent were then dropped on the cell suspension and plates centrifuged at 1,400 rpm for 7 minutes. For induction of NGN3, cells at day 11–12 of differentiation were lifted with TrypLE and replated in Aggrewells in 2.5 ml of medium in the presence of 10uM ROCKi. One hundred and fifty microliters of Opti-MEM medium containing NGN3-specific gRNA (gRNA#3, 1ug) and 5ul of lipofectamine RNAiMax transfection reagent were then dropped on the cell suspension and plates centrifuged at 1,400 rpm for 7 minutes. To minimize toxicity, culture media were replaced 8 hours after transfection. Induction of the transcriptional programs in non-engineered lines (e.g. H1 and CVI-A2 PSCs) followed similar protocols of cell lifting and replating except DOX was omitted. In this case, EBdCas9-mRNA (3ug) and gRNAs (1 ug) diluted in 125 ul Opti-MEMwere were combined in 125 ul Opti-MEM containing 5ul of Lipofectamine MessengerMax transfection reagent (Invitrogen, LMRNA003) and the mixture dropped onto the cells in 2.5 ml culture medium. Plates were then centrifuged at 1,400 rpm for 7 minutes and returned to the incubator for 8 hours, after which culture medium was changed.

### RNA extraction and RT-qPCR analysis.

RNA was extracted using RNAeasy (Qiagen) according to the manufacturer’s instructions. RNA samples were treated with Rnase-free DNase (Ambion), phenol-chloroform extracted, ethanol precipitated, resuspended in Rnase-free water and quantified using a Nanodrop ND-1000. Reverse transcription was performed using iScript (BioRad). 10 ng of cDNA was used to perform qRT-PCR using Sensimix SYBR Hi-ROX Kit (Meridian Biosciences) and an Applied Biosystems 7300 real-time PCR system. The following gene-specific primers were used: *PDX-1* Fw: CAACAAGTACATCTCACGGC; *PDX-1* Rev: CCTCCTCCTTTTTCCACTTCA; *NGN3* Fw: CTAAGAGCGAGTTGGCACTGA; *NGN3* Rev: GAGGTTGTGCATTCGATTGCG; *18S* Fw: GTAACCCGTTGAACCCCATT; *18S* Rev: CCATCCAATCGGTAGTAGCG; qRT-PCR conditions were: stage 1, 50°C for 2 min; stage 2, 95°C for 10min; Stage 3: 95°C for 15sec, 60°C for 30sec (40 Cycles). For each primer combination, amplification efficiency was consistently >95%. Threshold cycle numbers (*Ct*) were determined using the SDS 2.3 software (Applied Biosystems) and analyzed using the ΔΔ*Ct* method.

### Western Blotting analysis.

Whole protein extracts were prepared using 1X Cell Lysis Buffer (Cell Signaling Technology) (i.e., 1% TX-100, 20mM Tris pH7.5, 150mM NaCl, 1mM EDTA, 1 mM EGTA, 2.5 mM sodium pyrophosphate, 1 mM b-glycerophosphate, 1mM Sodium orthovanadate) in the presence of 0.1% SDS, a cocktail of protease inhibitors (Complete, Roche), and PMSF (1 mM). Protein concentration of lysates was determined by the BCA protein assay (Pierce). Thirty micrograms of protein were separated under reducing conditions onto 4%–12% polyacrylamide gels (Nu-Page, Invitrogen), transferred by Western blotting onto PVDF membranes (Immobilon, Millipore) and blocked in 5% BSA–0.1% Tween-20 overnight at 4°C. Blots were then incubated overnight at 4 C with the following primary antibodies: mouse anti-Cas9 mAb (Clone 7A9–3A3, Active Motif), rabbit anti-EZH2 (clone D2C9, Cell Signaling), rabbit anti-Histone H3K27me3 (clone C36B11, Cell Signaling), rabbit anti-Histone H3 (Clone 1B1B2, Cell Signaling), Rabbit anti-RFP (Rockland), mouse anti-alpha tubulin (ab7291, Abcam), mouse anti-PDX1 mAb (E-1, Santa Cruz Biotechnology), mouse anti-NGN3 (clone F25A1B3, DHSB). Secondary antibodies were HRP-conjugated donkey-anti mouse and donkey anti-rabbit Fab2 (Jackson Abs). Membrane-bound antibodies were detected by chemiluminescence using the WesternSure Chemiluminescence Reagent (Li-COR) and chemiluminescent signal recorded digitally using an Odyssey M Imager and ImageStudio software version 5.2 (LI-COR Biosciences).

### CUT&Tag assays.

Duplicate samples of stem cell clusters, induced to express EBdCas9 alone or PDX1/NGN3-guided EBdCas9, were harvested 48 hours after NGN3 gRNA transfection (day 14) and dissociated into single cells by TrypLE. Five hundred thousand cells per condition were then processed for CUT&Tag using the CUT&Tag-IT kit (Active Motif), as per manufacturer’s instructions. A Rabbit polyclonal specific for H3K27ac (Active Motif #39133) was used to isolate 300–500 bp chromatin fragments. DNA libraries were PCR amplified using a unique combination of i5/i7 indexing primers for each sample, cleaned using SPRI beads and pooled into an equimolar library for sequencing. Libraries were sequenced with an Illumina NextSeq 2000 (San Diego, CA) using XLEAP chemistry. More than 7 million paired end reads per sample were demultiplexed and converted to fastq format using BCL Convert v2.5.0 software through Illumina’s BaseSpace Sequencing Hub.

FASTQ files were trimmed with TrimGalore and mapped with Bowtie2 to hg38. Quality of files was assessed using FASTQC and MultiQc. Mapped files were converted to bed file using BEDTools^88^ prior to peak calling with SEACR^89^, selecting the top 95% of regions. Common peaks between replicates were defined as the union regions between replicates. Unique peaks were defined as non-overlapping peaks in one sample compared to another. HOMER^90^ was used to annotate peaks to nearest predicted gene target. BAM files were converted to bigwigs using DeepTools^91^ for visualization on UCSC Genome Browser. Heatmaps of enrichment were generated using DeepTools. The fastq files were submitted to SRA under the number PRJNA1406314. Reviewers can access them through the following link: https://dataview.ncbi.nlm.nih.gov/object/PRJNA1406314?reviewer=li82sp39itn1k39ujvvt3f6ats.

### Immunofluorescence and flow cytometry.

SC-derived islet clusters were dissociated into single-cell suspensions by TripLE treatment and fixed in BD Cytofix/Cytoperm^™^ solution for 20 minutes in ice. Cells were then washed in 1× BD Perm/Wash^™^ Buffer, pelleted, resuspended in BD Cytoperm^™^ Permeabilization Plus Solution and incubated for 10 minutes in ice. After washings in 1× BD Perm/Wash^™^ Buffer, samples were blocked with rabbit and mouse IgGs for 15 minutes in ice, and then stained with PE-conjugated rabbit monoclonal anti-Insulin (clone EPR17359, abcam #ab213192) and Alexa647-conjugated mouse anti-Glucagon antibody (Clone # 181402, R&D #IC1249R) diluted in 1x BD Perm/Wash^™^ Buffer for 1 hour at room temperature. After washings, cells were resuspended in HBSS/0.1%BSA and analyzed at a FACScalibur (Beckton Dickinson).

### Tissue Immunostaining and microscopy.

SC-derived islet clusters were fixed in 4% PFA overnight at 4 C and embedded in paraffin for histology. Seven-micron sections were cut and processed for immunofluorescence. Briefly, sections were first subjected to antigen retrieval by boiling in either citrate buffer (10 mM citrate 0.05% Tween20, pH 6.0) or 10 mM Tris Buffer, pH 9.00, for 30 minutes followed by cooling to room temperature for 1 hour. Sections were then blocked in 50 mM glycine for 10 minutes at room temperature, followed by incubation in PBS/1% BSA/2% donkey serum for 1 hour at room temperature. Tissue sections were then incubated overnight at 4°C with primary antibodies in blocking buffer. Primary antibodies included: guinea pig anti-insulin (A0564, Dako), mouse anti-glucagon (Sigma, clone K79bB10), Rabbit anti-Glucacon (Abcam, ab92517, rat anti-Somatostatin, R&D MAB2358), goat-anti-PPY (R&D AF6297), goat anti-PDX-1 (Abcam, ab47383), mouse anti-E-cadherin Ab (BD 610182, clone 36/Ecadherin), rabbit anti-Chromogranin (Abcam, ab45179), mouse anti-NKX6.1 (DSHB, clone F55A10), Rabbit anti-CDX2 (Abcam, ab76541)., Rat anti-SOX2 (eBioscience, clone Btjce) Binding of primary antibodies was revealed with Fab2-species-specific Alexa 647-, Rhodamine- and Alexa 488-conjugated donkey secondary antibodies. After staining, slides and coverslips were counterstained with DAPI, mounted and visualized either at a NIKON Eclipse-i90 or at a confocal NIKON A1R microscope equipped with a Spot II CCD camera. Morphometric analysis was performed on 10–12 sections per sample collected at approximately 50-μm intervals throughout each block, using the Spot Advanced and ImageProPlus software.

### Bulk RNAseq and analysis.

Bulk RNAseq was performed on differentiated MEL1-Double^FOXA2/INS^ and H1 hESC (day 20), induced to express EBdCas9, EBdCas9 guided sequentially by PDX1/NGN3 gRNAs or EBdCas9 guided by either PDX1 or NGN3 gRNAs. RNA was collected using an RNAeasy Minikit (Qiagen) and library preparation and sequencing was performed by Novogene (Sacramento, CA, USA). Briefly, mRNA was purified from total RNA using poly-T oligo attached magnetic beads. After fragmentation, the first strand cDNA was synthesized using random hexamer primers, followed by the second strand cDNA synthesis. Library was ready after end repair, A-tailing, adapter ligation, size selection, amplification, and purification. Samples were sequenced at an averaged 30 million raw reads using Illumina NovaSeq X Plus platform. Reads were aligned to the human reference genome hg38 using HISAT2^92^.

Gene expression analysis was performed using DESeq2 (v1.46.0) (doi:10.1186/s13059-014-0550-8.). Treatment (PDX1/NGN3-guided, NGN3-guided and PDX1-guided) vs control (EBdCas9 only) comparisons were performed for each cell line individually with an estimation of dispersion of 0.1. Protein-coding genes with at least 5 counts in one of the samples and with the same direction in their log_2_[fold change] for both cell lines were selected for further analysis. Gene set enrichment analysis was performed using clusterProfiler (v4.14.6) (https://doi.org/10.1089/omi.2011.0118) leveraging Reactome gene sets from MSigDB (msigdbr v25.1.1). Nominal enrichment P-value < 0.05 was used to designate significance. All RNA-seq data meeting MINSEQE (Minimum Information About a Next-generation Sequencing Experiment) guidelines have been deposited at Gene Expression Omnibus (GEO) with accession number GSE317542. Reviewers can access via the following token: ongpawgydrwbnkx.

### Tissue transplantation, in vivo Glucose Tolerance Tests and Insulin secretion.

Day 35–45 SC-derived islet clusters (about 500) were transplanted under the kidney capsule of immunodeficient NGS mice via a minimal (~ 5mm) skin incision on the left flank of the animal and exposure of the kidney. Tissues was then injected under the kidney capsule using a blunt plastic microcapillary. The muscle wall was sutured with absorbable sutures and the skin closed with non-absorbable sutures. At 3 and 6-months post-transplantation, glucose tolerance tests (GTT) were performed on 5 hours fasted animals by intra-peritoneal injection of a glucose solution (1.5 mg/g of body weight) and glycemia measured at 0’, 15’, 30’, 60’ 90’ and 120’ post glucose load. Blood glucose levels were monitored by tail prick using a FreeStyle glucose monitoring system (Abbott Diabetes Care Inc., Alameda, CA). Plasma levels of human C-peptide was measured at 0’ and 30’ post glucose load using an ultra-sensitive insulin human-specific ELISA kit (Alpco, Salem, NH).

### Statistics.

Statistical significance of differences among experimental groups was validated by 2-tailed Student’s *t* test or by ANOVA 1-group variance test for multiple comparisons, followed by Bonferroni post-hoc test using Prism Software (v10). Limit of significance was set at *P* < 0.05.

## Supplementary Material

Supplement 1
