# Supplementary material for: Epigenetic control of PDX1 and NGN3 by a computationally designed PRC2 inhibitor enforces pancreatic endocrine differentiation from pluripotent stem cells
Source: Res Sq. 2026 Mar 26:rs.3.rs-9115136. Preprint. [Version 1] doi: 10.21203/rs.3.rs-9115136/v1 (PMC13042171; doi:10.21203/rs.3.rs-9115136/v1)
Supplement: Supplement 1 [file NIHPPrs9115136v1-supplement-1.pdf]

Supplemental Figures.

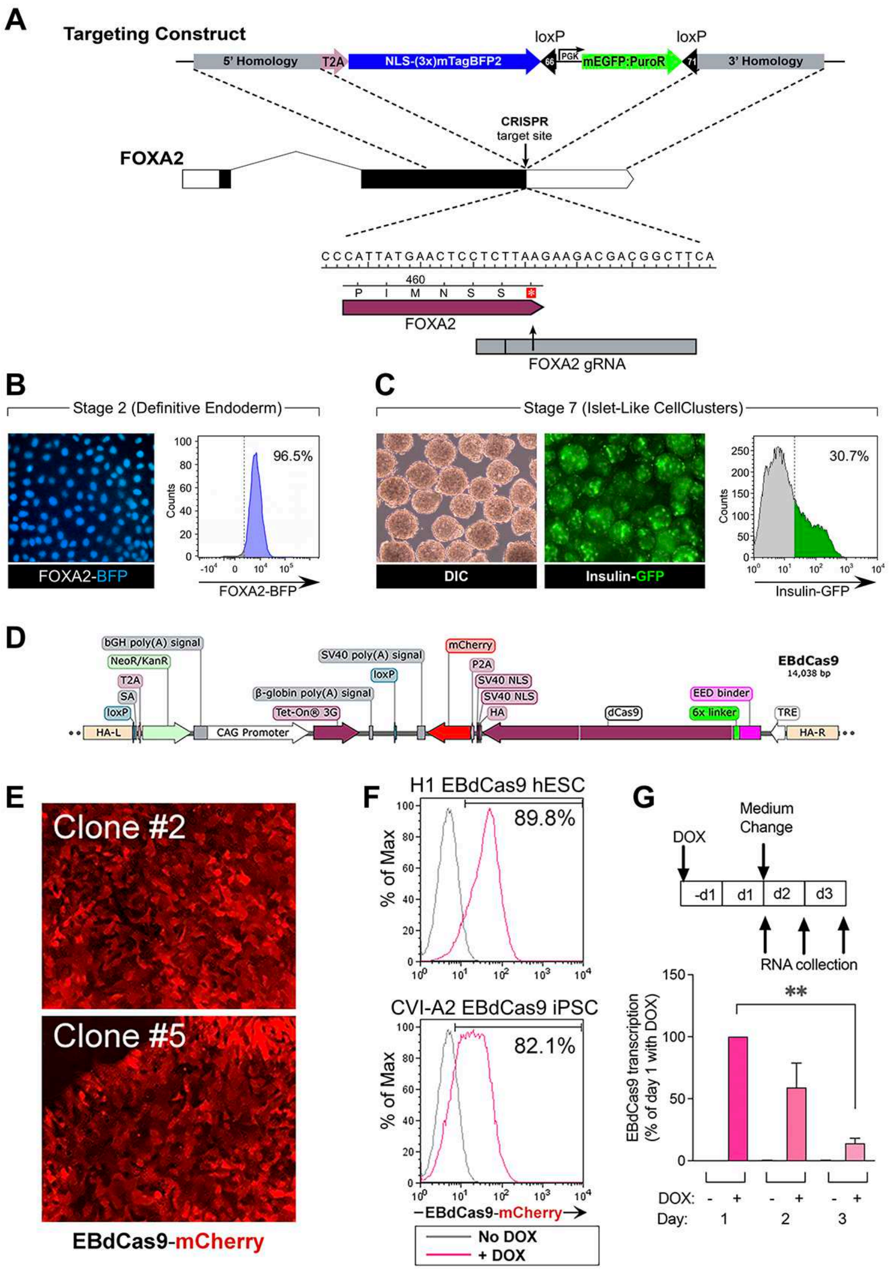

Figure S1. Generation of MEL1-Double$^{FOXA2/INS}$ reporter lines and engineering of the PRC2 EpiBinder inhibitor EBdCas9 into the AAVS1 safe harbor of hESC and iPSC lines. (A) Targeting construct used to target BFP downstream of the FOXA2 promoter via gRNA-guided CRISPR-mediated recombination. (B) Fluorescence microscopy image (left panel) and flow cytometry histograms (right panel) of BFP expressed in MEL1-Double$^{FOXA2/INS}$ cells differentiated to definitive endoderm stage, showing uniform FOXA2-driven expression of the fluorescent reporter in all cells. (C) Bright and fluorescence microscopy images (left panels) and flow cytometry histograms (right panel) of MEL1-Double$^{FOXA2/INS}$ cell clusters at stage 7 of differentiation (day 35), showing expression of the GFP reporter of insulin expression in ~ 30% of the cells. (D) EBdCas9-mCherry construct used to target the AAVS1 locus. (E-F) DOX-inducible expression of the construct in MEL1-Double$^{FOXA2/INS}$; EBdCas9^{mCherry} lines as detected by fluorescence microscopy (E) and flow cytometric analysis (F) of the mCherry reporter. (G) Timeline of DOX induction and qPCR analysis of EB-transcription in EBdCas9 reporter lines. ***$P<0.01$*

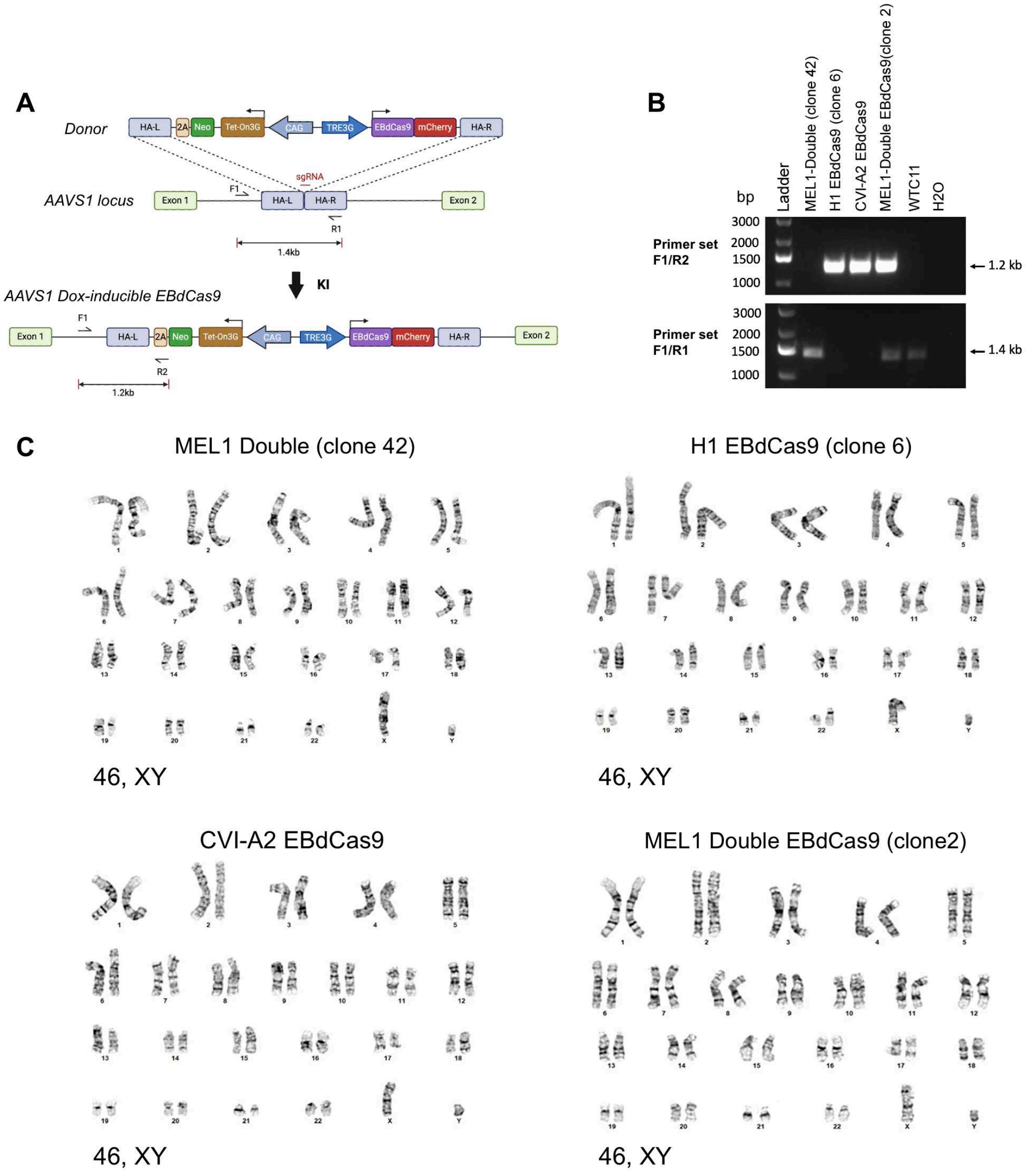

Figure S2. Validation of karyotypes and EBdCas9 genotypes of the engineered PSC lines. (A) Schematic diagram of targeting strategy for CRISPR-Cas9-mediated reporter gene knock-in at the human AAVS1 locus. The location of F1, R1 and R2 PCR primers used for genotyping is indicated. (B) PCR analysis of the EBdCas9 construct into the indicated engineered PSC lines to validate insertion into the AAVS1 site. The F1/R2 primer set was used to detect the insertion of the construct into AAVS1 locus (PCR amplicon: 1.2kb). The F1/R1 primer set was used to detect unmodified AAVS1 (PCR amplicon: 1.4kb). The co-amplification of the 1.2-and 1.4-kb fragments, or the amplification of the 1.4 kb fragment only, identify mono and biallelic insertion at the AAVS1 locus, respectively. (C) Karyotyping and G-banding analysis of MEL1-Double$^{FOXA2/INS}$, H1-EBdCas9 and CVI-A2-EBdCas9 lines. All tested lines are euploid and devoid of gross chromosomal aberrations.

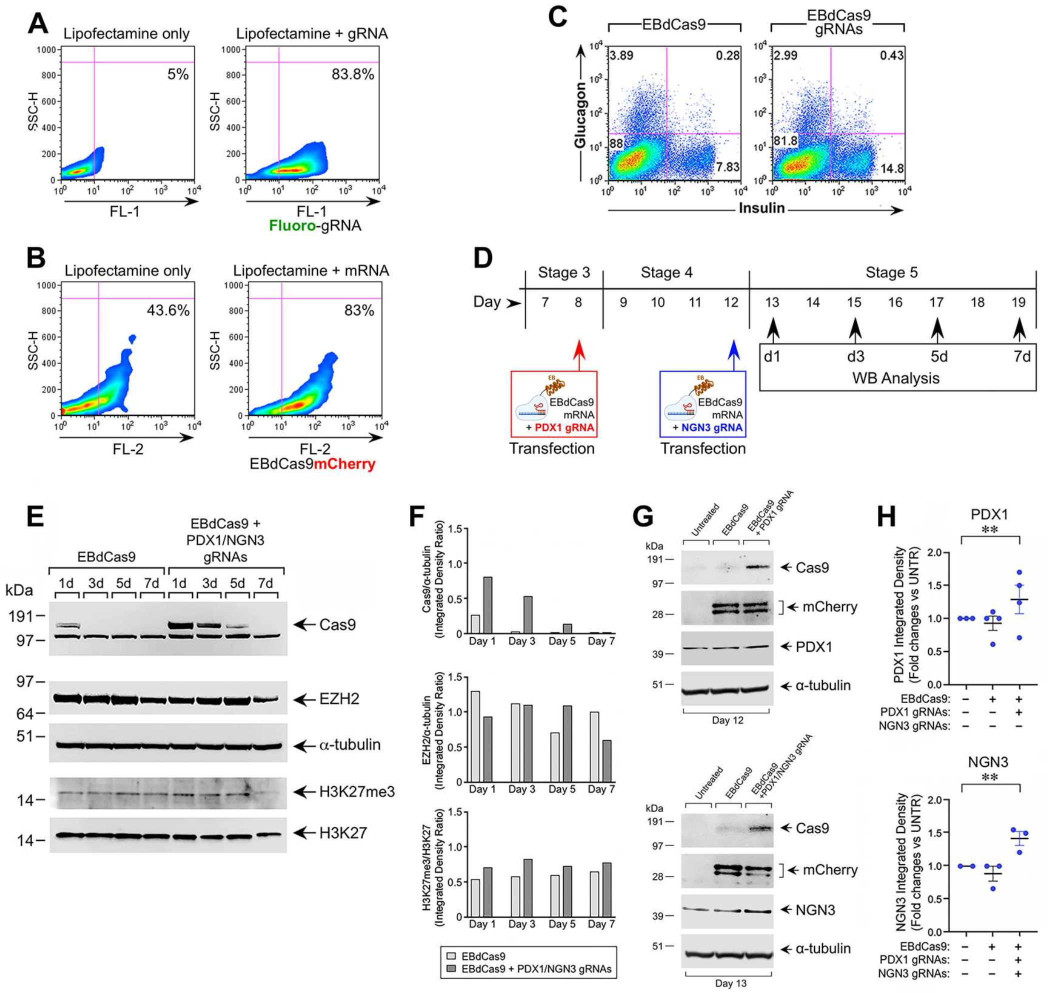

Figure S3. Transient expression of gene-guided EBdCas9 in non-engineered PSC lines is sufficient to increase β cell yields during PSC differentiation. (A-B) Representative flow cytometry plots showing the efficiency of Lipofectamine-mediated targeting of PSCs by fluorescinated gRNA (A) or dCas9-mCherry mRNA (B) as compared to lipofectamine-only controls. (C) Representative flow cytometric dot plots of PSCs transfected with EBdCas9 mRNA only or EBdCas9 mRNA and PDX1/NGN3 gRNAs as timed in Figure 4A, differentiated to day 19 and immuno-stained for insulin and glucagon. Gene-guided EBdCas9 drives a two-fold increase in the frequency of insulin$^+$ beta cells. (D) Timeline of Western blotting analysis of differentiating PSC samples. (E-F) Time course of Cas9, EZH2, H3K27me3, alpha-tubulin and total H3K27 expression detected by Western Blotting in whole cell extracts of differentiating PSC, 1 day up to 7 days after induction of EBdCas9 alone or EBdCas9+PDX/NGN3gRNAs. Expression of gRNA-guided EBdCas9 decreases progressively over 5 days in the targeted cells after gRNAs transfection, whereas minor changes in global expression of EZH2 and H3K27me3 are observed. (G) Western Blotting analysis of Cas9, mCherry, PDX1 and NGN3 proteins detected in whole cell extracts of the indicated samples 4 days (upper panel) and 1 day (lower panel) after transfections with EBdCas9mRNA+PDX gRNA or EBdCas9+PDX/NGN3 gRNAs, respectively. (H) Cumulative analysis of PDX1 and NGN3 protein levels normalized to alpha tubulin as detected by Western Blotting of whole cell extracts from 3-4 independent experiments, 4 days (PDX1) and 1 day (NGN3) after gRNA transfection. **=P<0.01.

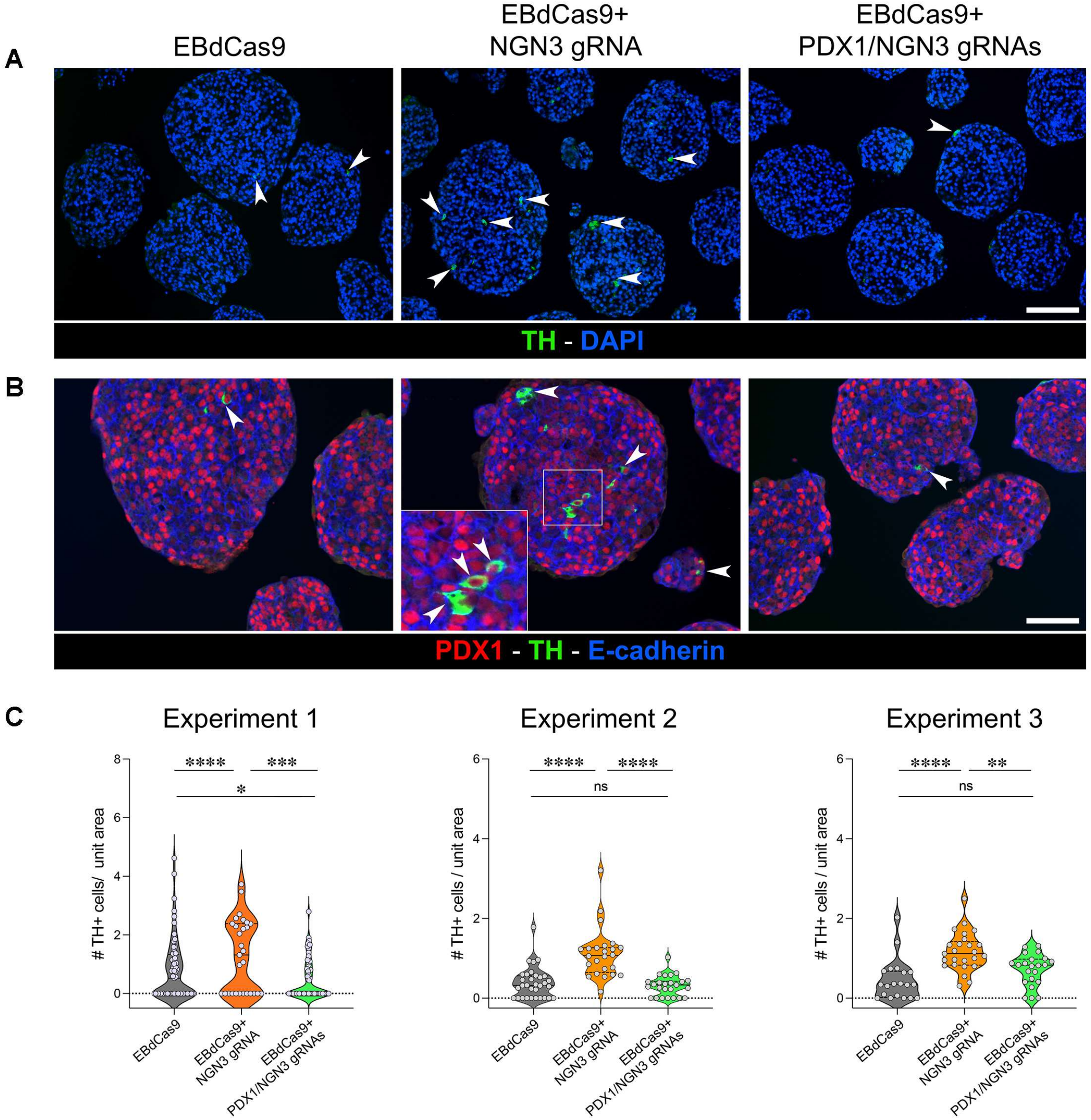

Figure S4. EBdCas9-mediated activation of *NGN3* promoter alone increases the frequency of entero-endocrine TH^+ cells. (A-B) Fluorescent images of PSC clusters at day 20 of differentiation from the indicated treatments, stained for Tyrosine-Hydroxilase (TH- green), a marker of enteroendocrine cells, PDX1 (red) and DAPI (blue), showing increased numbers of TH^+ cells in the clusters treated with NGN3-guided EBdCas9. (C) Violin plots showing the frequency of TH^+ cells counted in individual clusters in three independent differentiation experiments using H1 (Exp. 1) and MEL1-Double$^{FOXA2/INS}$ -hESC (Exp 2&3) PSCs. The thick black line across the violin plots is the median, and the dashed lines represent the SD **=P<0.01, ***=P<0.05, ****=P<0.001.

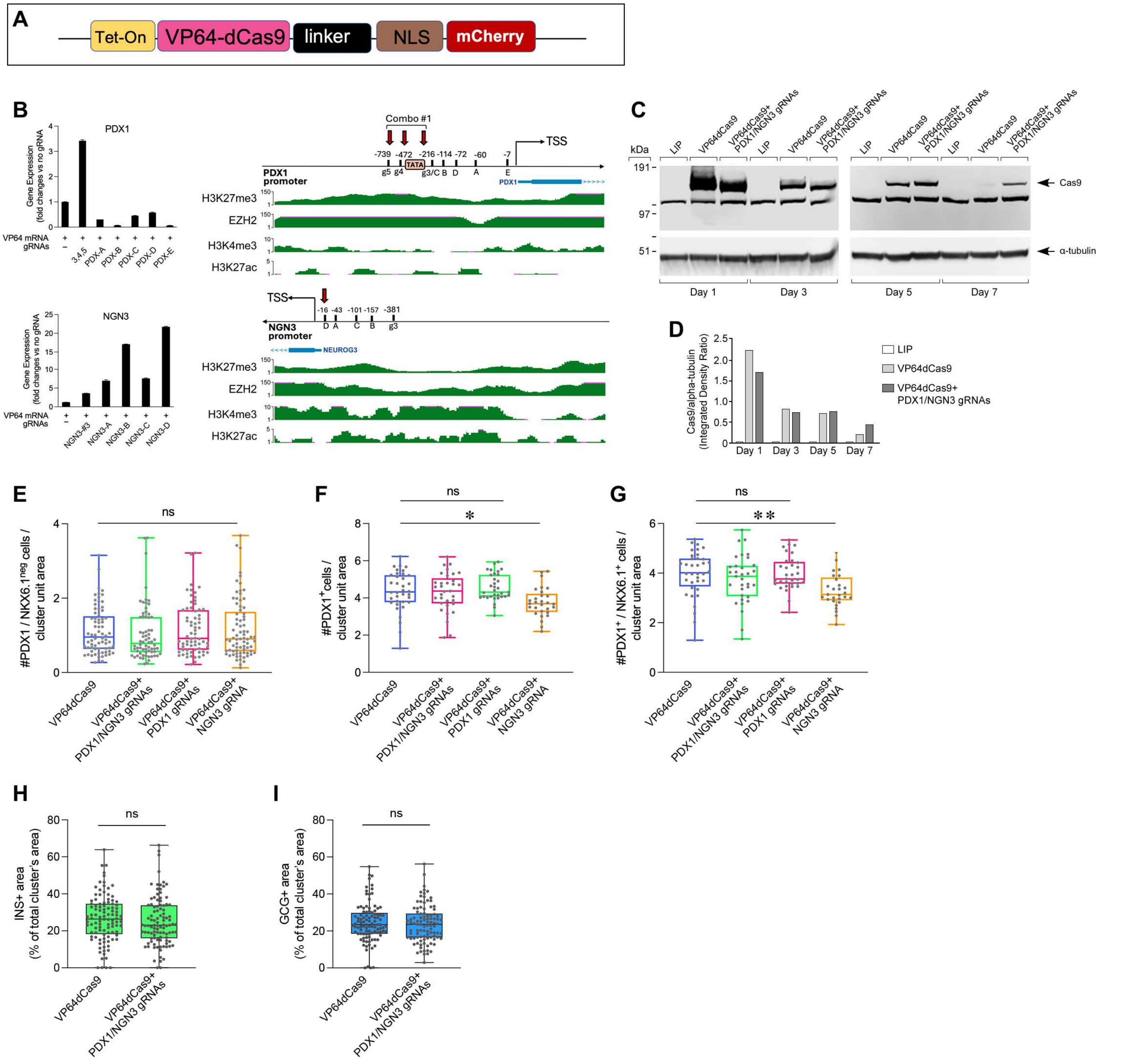

Figure S5. Gene activation by VP64 does not resolve clusters' heterogeneity nor it leads to increase β cell yields. (A) Construct used as template to synthesize VP64dCas9-mCherry mRNA. (B) Genomic coordinates of gRNAs designed for targeting the *PDX1* and *NGN3* promoters with VP64dCas9 and qPCR analysis of PDX1 and NGN3 transcription induced upon VP64dCas9-mCherry mRNA and gRNA transfection. (C-D). Western blotting analysis of Cas9 and alpha-tubulin (C) and relative quantification of protein-specific bands (D) in whole cell extracts of differentiating MEL1-Double$^{FOXA2/INS}$ -hESC, 1 day up to 7 days after lipofectamine treatment only (LIP), lipofectamine-mediated transfection of VP64dCas9 mRNA alone or VP64dCas9+PDX/NGN3gRNAs. Expression of gRNA-guided VP64dCas9 decreases progressively overtime but it is still detectable 7 days post-transfection. (E-G) Morphometric analysis of cell clusters at day 20 of differentiation, showing the frequency of uncommitted PDX1^{neg}/NKX6.1^{neg}, total PDX^{+} and PDX^{+}/NKX6^{+} cell types normalized to clusters' areas under each treatment. (H-I) Box-and-whiskers-plots showing the distribution of insulin and glucagon-positive areas measured in individual clusters at stage 7 (day 35) generated from 2 differentiation experiments using MEL1-Double$^{FOXA2/INS}$ -hESC.

Supplementary Files

This is a list of supplementary files associated with this preprint. Click to download.

- TableS8.xlsx
- TableS9.xlsx
- TableS11AD.pdf
- TableS10.pdf
- TableS7.xlsx
- TableS6.xlsx
- TableS3.xlsx
- TableS4.xlsx
- TableS2.xlsx
- TableS5.xlsx
- TableS1.xlsx
